# Domain-specific effects of hunger on attention and choice

**DOI:** 10.1038/s41598-026-48772-0

**Published:** 2026-04-21

**Authors:** Jennifer March, Chih-Chung Ting, Soyoung Q. Park, Sebastian Gluth

**Affiliations:** 1 Department of Psychology and Hamburg Center of Neural and Cognitive Systems, University of Hamburg, Von-Melle-Park 11, 20146 Hamburg, Germany; 2 Department of Decision Neuroscience & Nutrition, German Institute of Human Nutrition Potsdam-Rehbrücke, 14558 Nuthetal, Germany; 3 German Center for Diabetes Research, 85764 München-Neuherberg, Germany; 4 Charité-Universitätsmedizin Berlin, Corporate Member of Freie Universität Berlin, Humboldt-Universität Zu Berlin and Berlin Institute of Health, Neuroscience Research Center, 10117 Berlin, Germany

**Keywords:** Eye-tracking, State change, Computational modeling, Neuroscience, Psychology, Psychology

## Abstract

Hunger leads to less healthy food choices, but its effect on decision-making in non-food domains is less clear. To close this gap, we investigated to what extent hunger state affected choice across domains by examining attentional patterns and cognitive mechanisms underlying decision-making across different domains. We implemented a within-subject design in which participants completed a food choice, an intertemporal discounting, and a social preferences task in hungry and sated states while their eye-movements were recorded. Hungry participants were more likely to look at and subsequently select tasty over healthy food items, while attention and choice remained unaffected in the non-food tasks. Cognitive modelling indicated that hunger state selectively affected attention-driven evidence accumulation processes in the food choice task. Altogether, our results are consistent with a domain-specific view, indicating that attentional and decision-making processes in non-food domains remain unaffected by states of elevated hunger.

**Supplementary Information:**

The online version contains supplementary material available at 10.1038/s41598-026-48772-0.

## Introduction

Hunger is among the most common physiological states people experience every day, whether due to meal skipping, time constraints or intentional temporal or caloric restrictions. Importantly, hunger has been linked to a decreased likelihood of healthy food choices^1–4^. One identified key driver of this effect is attention^2^, which also predicts decision-making more broadly^5–9^. While the effects of hunger state on attention and food choice are well-established, the impact of hunger state on attention and choice in non-food domains is much more limited. Given how commonly people experience hunger in daily life, it is important to understand how this physiological state affects choice processes across different domains to predict broader behavioral consequences. Therefore, the present study uses cognitive modeling of eye movements and decision-making behavior to examine the effect of hunger state on cognitive processes underlying choice across domains.

There is ample evidence for the effect of hunger state on food choice. An early study^10^ found that hungrier participants bought more food items in a grocery store. Extending this finding, more recent studies have shown that hunger state increases preference for immediate available food options^1,3^, larger portion sizes^1^ as well as calorie-dense, tasty and higher wanted food options^2^. These findings can be explained by the concept of energy restoration, wherein food deprivation creates a physiological imbalance which initiates regulatory mechanisms to reestablish homeostasis. While this mechanism offers a plausible explanation for the effects of hunger state on food choice, it cannot straightforwardly be applied to non-food choice domains. Nevertheless, there is evidence suggesting an effect of hunger state on choice across domains: In the intertemporal choice domain, evidence suggests that hungry individuals are more likely to discount future rewards across three choice domains^11^. More specifically, the researchers find that hunger state induces a high discounting of food options, which spills over to other choice domains. In the social domain, the evidence for an effect of hunger state on choice is mixed. While studies have suggested that hunger leads to more selfish behavior^12–14^, a more recent study^15^ portraits a more nuanced view: Across five studies, the authors show that there is only a small increase of hunger on selfish behavior in non-interdependent tasks (i.e., tasks in which the choice outcome is exclusively determined by one decision maker, such as the dictator game) and no effect in interdependent tasks. In sum, the evidence indicates that hunger state increases the probability of what we term “hot” choices^16,17^. That is hungry participants are not only more likely to choose tasty over healthy options but also choose more impatiently and selfishly (with the across-domain spillover effects being smaller than the within-domain effect on food choice).

The impact of hunger on choice may stem from differences in how hungry individuals attend to and process information relevant to their decisions. Previous research has demonstrated a robust association between attention and choice across choice domains. That is, participants tend to choose the options they look at longer^5,8,18^, including meta-analytic evidence for a causal effect of attention on choice^9^. Physiological states such as hunger may also influence preference and attentional allocation. Within-domain studies suggest hunger state to induce an attentional bias toward food images in general^19,20^ and those of higher caloric density in particular^21^. Numerous studies have shown that participants instructed to focus on health are more likely to make healthy decisions, whereas taste-primed participants are more likely to make tasty decisions^22–27^. Such influences of instructions on choice behavior have also been shown in non-food domains, with participants allocating more attention to options that are more congruent with their current choice goal^28,29^. While to the best of our knowledge, the effect of hunger state on attentional allocation across choice domains is unclear, previous work explained an increase in the acquisition of non-food options in hungry participants by a general shift in the acquisition goals^30^. Thus, we also expect goal-congruent shifts in attentional allocation under hunger. In sum, the current evident suggests that the relationship between attention and choice is domain-general, and that hunger state may shifts attention toward hot options leading to an increase in hot choice across domains.

Computational models in decision-making, such as the Diffusion Decision Model^31^(DDM), are used to describe and explain the mechanisms underlying choice and predict behavioral patterns. They offer a principled account to test domain-general and -specific effects. DDMs assume that during decision-making, people continuously sample evidence about the appeal of the options. The average rate at which evidence in favor of an option accumulates is called the drift rate. A choice is made, when the accumulated evidence reaches a threshold. Multi-attribute DDMs allow for distinct impact (i.e., weight) of the options’ underlying attributes (e.g., taste and health)^32^. Importantly, various multi-attribute DDMs have been used to explain food^2,33,34^, intertemporal^35^ and social choice^36,37^, suggesting that a common mechanism of evidence accumulation that trades off multiple attributes (i.e., taste vs. health, delay vs. amount, self vs. other) underlies these types of value-based decisions. More recent DDMs also include attentional mechanisms, taking into account how the proportion of dwell time on one vs another option influences choice. These models assume that gaze has an amplifying effect on the drift rate^5,6^. Comparing the effects of hunger on model parameters across domains could provide critical insights on common and distinct underlying mechanisms. Candidate parameters that could be affected by hunger are (1) a starting point bias, (2) the weighting of attributes, or (3) attentional parameters to discount non-fixated options or attributes. First, in line with the idea that hunger state sets a prior preference towards one option at the beginning of the decision process. A recent study^38^ demonstrated that a placebo drink which participants expected to increase (decrease) hunger induced a starting point bias to accept (reject) food options. Similarly, hunger may bias participants prior to the choice process to make more “hot” decisions (i.e., more tasty, impatient, selfish) across domains. Second, a hunger-induced shift in attribute weights aligns with work^29^ showing how instructions changed choice goals (i.e., focus on health vs focus on taste), which in turn affected attribute weighing in the goal-congruent direction. Following this, the effect of hunger state on choice across domains could be explained by altered goals which affect the choice process through the importance assigned to the underlying attributes. Third, in line with an attentional discounting effect, we have previously shown that hunger state influenced attentional discounting of health information (i.e., cold attribute) in a food choice task^2^. Therefore, the effect of hunger state on choice may increase participants’ attentional discounting of cold attributes leading to an increase in hot decisions across domains. In sum, computational modelling will allow us to determine the mechanisms through which hunger state affects choice across domains.

The goal of the present study was to test and mechanistically understand the effects of hunger state on attention and choice across domains. To this end, we conducted a within-subject experiment in which participants completed three different choice tasks (food, intertemporal, social) once in hungry and once in sated states, while we recorded their eye movements. In addition, we use cognitive modelling to explain how hunger state influences the interaction of attentional allocation and preference formation. We expected hunger to increase hot decisions across choice domains (more tasty, impatient and selfish decisions), resulting from an attentional shift. In addition, we predicted that these differences would manifest in our computational model through consistent modulation of the same parameters across choice domains. Our behavioral, eye-tracking and modelling results suggest that the effect of hunger state on attention and choice is domain-specific: Behaviorally, we show that hunger state increased tasty, but not impatient and selfish choice. Our eye-tracking analyses reveal that hungry participants were more likely to look at hot options, which in turn increased their probability of choice only in the food but not in the intertemporal and social domain. Convergingly, our cognitive model demonstrates hunger-driven differences in the weighting of attributes and attentional discounting parameters exclusively in food-related decisions.

## Method

### Participants

In line with our a priori power analysis (for details see ref. 6 or the preregistration protocol at osf.io/tmdw3), our sample size consisted of 70 participants (53F, 16 M, 1D) with a mean age of 25.6 (${SD}_{age}$=8.064) and a mean BMI of 23.22 (${SD}_{BMI}$=4.363). Most participants (*n* = 40) were psychology students from the University of Hamburg, others (*n* = 30) were recruited using the online platform Stellenwerk (stellenwerk-hamburg.de). Eligibility criteria were proficiency in German and a minimum age of 18 years. Exclusion criteria were dietary restriction, food allergies or intolerances, physical or mental illnesses, drug use, as well as pregnancy and breastfeeding. The study was approved by Local Ethics Committee of the Faculty of Psychology and Human Movement Sciences at the University of Hamburg (AZ: 2022_010) and was carried out in accordance with the institution’s guidelines and regulations. All hypotheses and analyses were preregistered on Open Science Framework (osf.io/tmdw3).

### Design and procedure

**Fig. 1:**
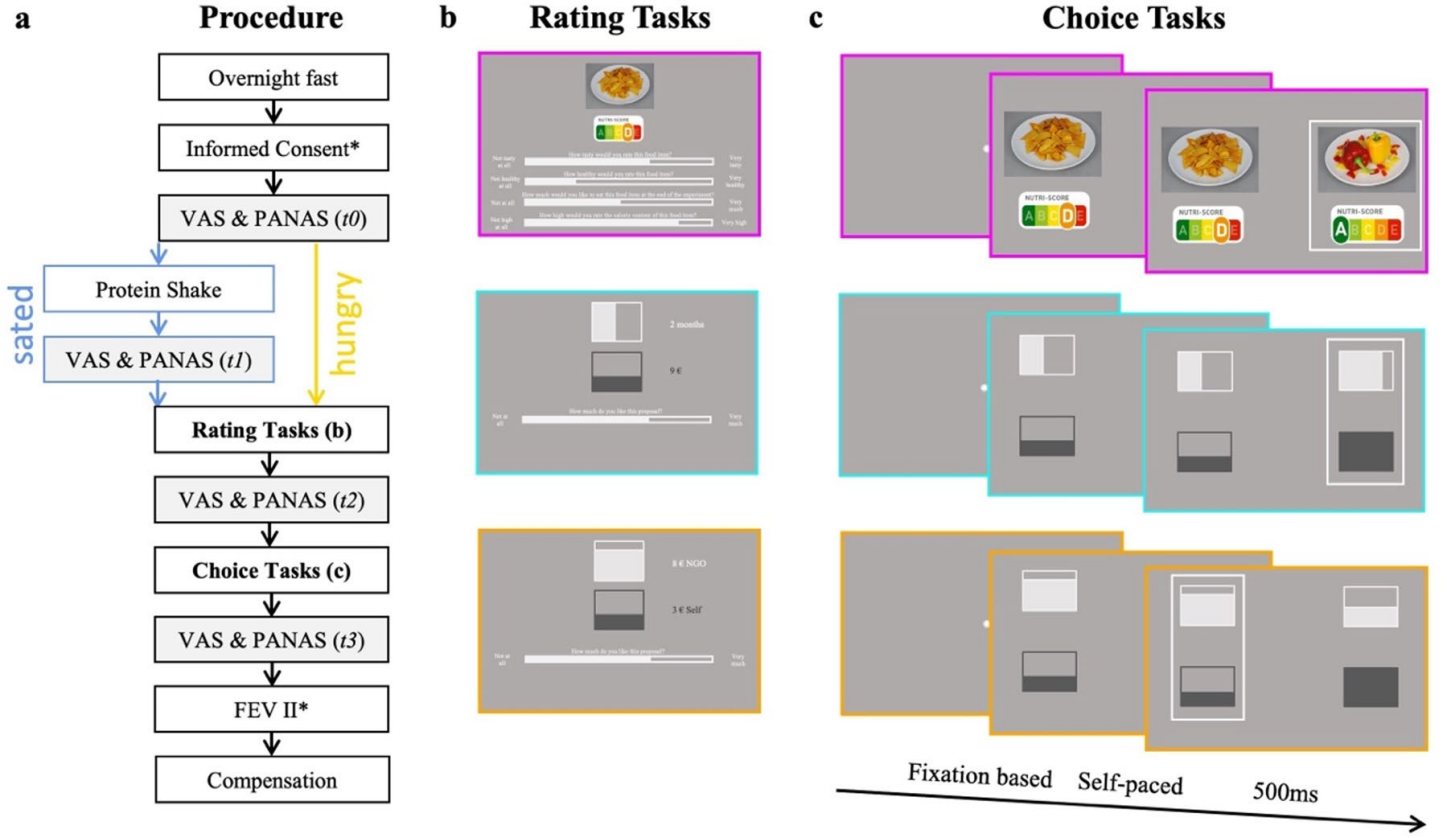
Rating and choice tasks across three domains. (**a**) Within-subject design, in counterbalanced order: Participants arrived at the lab after an overnight fast. In the sated (*blue*) condition, they received a protein shake, and in the hungry condition (*yellow*) they did not receive anything; hunger state (*VAS*) and mood (*PANAS*) were assessed at several timepoints (*t0*, *t1*, *t2* and *t3*) throughout each session, participants completed three rating tasks (**b**) and three choice tasks (**c**) in counterbalanced order and finally indicated their eating behavior (*FEV II*), * indicates that this step was only implemented in the first session (here: Informed Consent and FEV II); (**b**) Rating tasks, participants rated food items (*pink frame;* here: nachos with Nutri-Score D) in terms of tastiness, healthiness, wanting and perceived caloric content; temporal offers (*turquoise frame;* here: €9 book store coupon in 2 months) and social offers (*orange frame*; here €8 to NGO and €3 to self) were rated in terms of liking (**c**) binary choice tasks with eye-movement recordings, food choice tasks (*pink frame*) included tasty vs healthy food options; intertemporal choice task (*turquoise frame*) included sooner smaller vs later larger rewards and social choice task (*orange frame*) included prosocial (i.e., small amount to self and large amount to NGO) vs selfish (i.e., large amount to self and small amount to NGO) options.

We used a 2 × 3 fully within-subject repeated measures design to investigate the effect of hunger state (hungry vs. satiated) across three domains of value-based decision-making (food, intertemporal discounting and social). Each participant completed all three tasks in hungry and sated states respectively. Condition order was counterbalanced, resulting in 34 participants who were tested in the hungry condition first and 36 who were tested in the sated condition first. There were five to ten days between sessions. In both conditions, participants were instructed to fast overnight and refrain from eating in the morning before the experiment. While participants did not receive any food in the hungry condition, they received a protein shake amounting to 25% of their daily caloric needs based on age, gender, weight and fitness level^39^ in the sated condition. At the beginning of the first session, participants read the instructions and gave their informed consent. In addition, participants could select one of three suggested Non-Governmental Organizations (NGO) for later incentivization of the social choice task (see *Incentivization*). The experiment was caried out in the eye-tracking lab of the Cognitive Modelling and Decision Neuroscience group at the Department of Psychology of the University of Hamburg using a 24-inch screen with a resolution of 1024 × 768 pixels. At multiple points during each session, participants rated their hunger levels and mood, to validate the manipulation and minimize potential confounds. The experiment consisted of three rating blocks and three corresponding choice blocks (food, intertemporal discounting and social) in counterbalanced order, with choice blocks matching the rating block sequence. Block order remained constant for each participant across both experimental sessions. We also controlled for eating behavior (*FEV II*; see 2.3 *Control Measures*) at the end of the first session. One session lasted approximately 2 h, at the end of which participants were compensated with course credit or money (€12,50/h) and received a bonus based on their choices (see 2.3.5 Incentivization, Fig. 1a).

### Materials

#### Food tasks

For both food tasks we used 66 standardized food items displayed on plates of the Full4Health Image Collection^40^ (available at osf.io/cx7tp/) and their corresponding Nutri-Score, which represents a color-coded nutritional rating of a food item within a food category (from green A = balanced to red E = unbalanced nutrition; Federal Ministry of Food and Agriculture). For more details on the selection of images see ref^2^..

The *food rating task* was used to determine the values of the later displayed choice options. Participants rated all 66 food images with respect to taste, health, wanting and caloric content. Each trial started with a fixation dot (1000 ms) followed by the rating screen. The upper part of the rating screen consisted of food image and corresponding Nutri-Score in counterbalanced vertical positioning across participants, and the lower part consisted of continuous white rating scales. Taste and health were rated from “not at all tasty/ healthy” to “very tasty/ healthy”. The extent to which participants would like to eat the displayed option reflected “wanting” (range: “not at all”; “very much”). Estimated caloric content ranged from “not high at all” to “very high”. Mouse movements modified the scale’s fill from its central starting point with ratings confirmed by a left mouse button press (Fig. 1b).

In the *food choice task* each option consisted of two attributes: taste as represented by food image and health as represented by the corresponding Nutri-Score. Participants made 190 food decisions divided into two blocks of equal length while their eye-movements were recorded. Before the start of the task and between blocks, the eye-tracker was (re-)calibrated. Each trial started with a fixation dot, which had to be fixated on for at least 1000 ms before the choice screen appeared. The choice screen (self-paced) was followed by a feedback screen showing the selected option in a black frame (500 ms) (Fig. 1c). Participants selected the option they preferred using left and right arrow keys. The food choice task lasted for approximately 25 min.

#### Intertemporal discounting tasks

In the intertemporal discounting tasks, attributes were the monetary amount of a book coupon and the delay at which it would be received. The distributions were displayed in vertically filled squares representing the amount and the squares for delay were horizontally filled (i.e., empty square = €0/ 0 month; full square = €10/ 6 month)^41^. Participants were informed about the representation of the distributions. The colors of the squares were dark grey (amount) and light grey (delay), with counterbalanced screen positioning in both rating and choice task.

The *rating task of the intertemporal discounting* was used to familiarize participants with the representation of the attributes. Overall, participants rated 10 trials with varying delay and monetary amounts for a book coupon. Delay ranged from immediately to six months in steps of one month. The value of the coupon ranged from €1–20 in steps of €1. The proposals ranged from patient (i.e., high amount and delay) to impatient (i.e., low amount and delay) and were fixed across participants. Next to the squares participants saw the amount in euros and the delay in months. Each trial started with a fixation dot (1000 ms) followed by the rating screen. The upper part of the rating screen consisted of vertically filled squares indicating amount and delay in counterbalanced vertical positioning across participants, and the lower part consisted of continuous white rating scales. Participants indicated how much they liked the distribution on a continuous scale from “not at all” to “very much”. Mouse movements modified the scale’s fill from its central starting point with ratings confirmed by a left mouse button press (Fig. 1b).

In the *intertemporal discounting choice task* each option consisted of an attribute representing amount and delay as displayed by dark and light grey filled squares, repectively. As in the rating task, amount refered to the value of the book coupon, while delay refered to the time at which the coupon would be received. Importantly, one option offered a higher amount to be received after a longer delay, while the other option offered a smaller amount to be received after a shorter delay. Participants made 190 intertemporal decisions divided into two blocks of equal length while their eye-movements were recorded. Before the start of the task and between blocks, the eye-tracker was (re-)calibrated. Each trial started with a fixation dot, which had to be fixated on for at least 1000 ms before the choice screen appeared. The choice screen (self-paced) was followed by a feedback screen showing the selected option in a black frame (500 ms) (Fig. 1c). Participants selected the option they preferred best using left and right arrow keys. The intertemporal choice task lasted for approximately 25 min.

#### Social tasks

In the social tasks, stimuli were monetary distributions between self and a self-selected NGO. As in the intertemporal discounting tasks the distributions were represented using vertically filled squares, where an empty square indicated €0 and a full square indicted €10^18^. Participants were informed about the representation of the distributions. The colors of the squares were dark grey (self) and light grey (NGO), with counterbalanced screen positioning in both rating and choice task.

The *social preference rating task* was used to familiarize participants with the representation of the attributes. Participants rated 10 trials with varying monetary distributions between themselves and the NGO of their choice. Each fund ranged from €0–10, in steps of €1, and the distributions could be selfish (i.e., larger amount to self), fair (i.e., same amount to self and NGO) or prosocial (i.e., larger amount to NGO). We did not fix the total value of the proposal. Next to the squares participants saw the funds for self and NGO in euros. Each trial started with a fixation dot (1000 ms) followed by the rating screen. The upper part of the rating screen consisted of vertically filled squares indicating the amounts for self and NGO in counterbalanced vertical positioning across participants, and the lower part consisted of continuous white rating scales. Participants indicated how much they liked the distribution on a continuous scale from “not at all” to “very much” (Fig. 1b). Mouse movements modified the scale’s fill from its central starting point with ratings confirmed by a left mouse button press.

In the *social choice task* each option consisted of a selfish and a prosocial attribute as represened by dark and light grey vertically filled squares, repectively. The selfish attribute indicated the amount of money (in euro) distributed to the participant, while the prosocial attribute indicated the amount distributed to the previously selected NGO^42–44^. Importantly, one option offered a higher allocation to self and the other a higher allocation to NGO. In other words, one option was more selfish, while the other was more prosocial. The total value of each options was not fixed. Participants made 190 social decisions divided into two blocks of equal length while their eye-movements were recorded. Before the start of the task and between blocks, the eye-tracker was (re-)calibrated. Each trial started with a fixation dot, which had to be fixated on for at least 1000 ms before the choice screen appeared. The choice screen (self-paced) was followed by a feedback screen showing the selected option in a black frame (500 ms) (Fig. 1c). Participants selected the option they preferred best using left and right arrow keys. The social choice task lasted for approximately 25 min.

#### Control measures

We controlled for *subjective hunger state* using a Visual Analogue Scale (*VAS*)^45^ on which subjects indicated how hungry and how satiated they felt at several timepoints throughout the experiment. The continuous scale ranged from “not hungry at all” to “very hungry” and from “not satiated at all” to “very satiated”. VAS scores are a reliable measure for appetite research^46^. Moreover, we controlled for *mood* using the German version of the *Positive and Negative Affect Schedule* (*PANAS*)^47,48^. The measure consists of 10 adjectives assessing positive (e.g., “interested”) and negative affect (e.g., “nervous”), respectively. On a 5-point Likert scale (1 = “not at all”; 5 = “extremely”), participants rated to what extent the adjective matched their current subjective mood. This measure demonstrates good reliability (Raykovs *p* > 0.9) and validity^47^. Mean scores of each subscale were used for exploratory analyses.

To control for *eating behaviour*, we used an abbreviated German version of the Dutch Eating Behavior Questionnaire^49,50^. The questionnaire includes three subscales which measure restrained eating (e.g., “I think about my weight when deciding what to eat”), emotional eating (e.g., “When I feel lonely, I want to eat”) and externalized eating (e.g., “I eat more than usually when I see others eating”), respectively, each using ten items. Items are rated on a five-point Likert scale (1 = “never”; 5 = “very often”). The three subscales demonstrate good reliability (restraint: α = 0.92, emotionality: α = 0.94, externalizing: α = 0.89) and construct validity^51^. Mean scores of each subscale were used for exploratory analyses.

At the beginning of each session, we collected information about participants last meal (time of consumption; meal size; description of content), their general breakfast habits (on how many days per week do participants eat breakfast and content of usual breakfast). Female participants were also asked to indicate their time of menstrual cycle.

#### Incentivization

We incentivized choices to ensure accurate responses in the choice tasks. In each session the food choice task and randomly one of the other choice tasks was incentivized. For food choices, one randomly drawn choice, for which the food item was rated higher than 50 out of 100 on wanting was incentivized. For the other two choice tasks one option that the participants selected in the choice task was incentivized: In case of the intertemporal discounting task, participants received a coupon of a local bookstore of the monetary amount and the temporary delay. In case of the social choice task, both participant and NGO received the money of the chosen distribution. The NGOs were collectively paid after data collection.

### Eye-tracking

An SR Research EyeLink 1000 Plus eye-tracker with up to 2 kHz sampling rate was used to record participants’ eye-movements during the three choice tasks. To avoid excessive head movements a chin rest was used in approximately 93 cm distance to the screen. For each choice task calibration was done at the beginning and after completing half of the trials. The eye-tracking data was preprocessed in Matlab (2021b, mathworks.com) using the edfmex converter (SR Research Ltd.). We parsed the events into trials and locations. Fixation areas of interest (AOI) were the positions on the screen at which the four attributes in each choice task with a margin of 5% were displayed.

### Data analysis

The aim of the data analysis is to reveal, whether and how hunger state affects choice across domains. While some results of the food choice task have already been reported^2^, it is important to repeat them here to facilitate comparisons between tasks for testing domain-specific vs. domain-general effects of hunger.

#### Behavioral analysis

Here we tested the hypothesis that hunger state affects choice and RT across domains (H1). Specifically, we predicted that hungry participants are more likely and faster to make tasty over healthy food choices (H1a) and that this effect spills over on intertemporal and social decisions. That is, hungry participants discount future rewards faster and more strongly (H1b) and are more likely and faster to make selfish decisions in non-interdependent social choice tasks (H1c). (We deviate here from our preregistered hypothesis to improve clarity of the manuscript and comparability of the effect of hunger state across choice domains. The preregistered hypothesis, which was not confirmed by the data, was that hunger state may increase participants reliance on their social preference, that is, under hunger selfish individuals would become more selfish, while prosocial individuals would become more prosocial. We tested this prediction as well, and the results are reported in Fig. S6).

All behavioral and modeling analyses were carried out in R (version 4.3.1). For the preprocessing of the behavioral data, trials that were 4 SD above the participant’s mean RT on each choice task, or less than 250 ms were excluded. We used t-tests to test the effectiveness of our hunger state manipulation and used Bonferroni correction to account for multiple tests. First, we performed an independent t-test at lab arrival to compare hunger ratings across sessions. Second, we used a paired t-test to assess the difference in hunger ratings before and after the consumption of the protein shake. Third, we used a paired t-test to assess whether hunger ratings changed throughout the hungry session. Fourth, we tested differences in hunger ratings between conditions after the consumption of the protein shake in the sated condition and before the beginning of the choice task in the hungry condition (Fig. S1a). In a similar way we also assessed the effect of our manipulation on mood (Fig. S1b, c). Ratings were normally distributed and did not contain extreme outliers. The sample of the manipulation check included only 64 participants, as six participants did not fill in VAS and PANAS before the administration of the protein shake. We report *t*-statistics, *p*-values and effect sizes based on Cohen’s *d*.

To test the effect of hunger state on choice and response times (RT) across domains (H1), we used Generalized Linear Mixed Effects Models (GLMM) using lme4^52^. First, for the choice GLMMs we used a mixed-effects logistic regression with binary outcome (hot vs cold): tasty vs. healthy (food domain), impatient vs. patient choice (intertemporal domain) and selfish vs. prosocial (social domain). We included condition (hungry vs. sated) and relative dwell time (DT) on the hot option (i.e., tasty, impatient, selfish), as well as DT on the hot attribute as predictors. As DT proportions were excessively larger on food images vs Nutri-Scores, we used a binary predictor of whether the nutritional score was looked at versus not to reflect attribute-specific attentional influence on food choice. For the non-food tasks, in which DT was more evenly distributed between attributes, we used the relative DT difference between hot and cold attributes. We used a binomial distribution with a logit link function. The predictors of the RT GLMMs were condition, the scaled absolute value difference of the hot and cold attributes and their interaction with condition. Here a gamma distribution with an identity link function was specified. For both choice and RT GLMMs, we found that models which included a random intercept for participants and a random slope for condition (${AIC}_{food}$ = 12,860.8; ${AIC}_{discount}$ = 23,028.41; ${AIC}_{social}$ = 23,610.1; ${AIC}_{food(RT)}$ = 24,438.93; ${AIC}_{discount(RT)}$ = 60,206.46; ${AIC}_{social(RT)}$ = 58,484.81) performed better than those without (${AIC}_{food}$ = 13,055.59; ${AIC}_{discount}$ = 28,294.36; ${AIC}_{social}$= 32,554.49; ${AIC}_{food(RT)}$ = 28,544.95; ${AIC}_{discount(RT)}$= 78,591.49; ${AIC}_{social(RT)}$= 75,315.87) or those that only included a random intercept (${AIC}_{food}$ = 13,660.96; ${AIC}_{discount}$ = 23,781.05; ${AIC}_{social}$ = 25,335.53; ${AIC}_{food(RT)}$ = 24,556.97; ${AIC}_{discount(RT)}$= 64,928.95; ${AIC}_{social(RT)}$= 61,118.03). Exploratory GLMMs which included other measures, such as mood, eating behavior and demographics are reported in the supplements (Tables S3, S7 and S11). We report correlation coefficients, standard errors (*SE*), as well as *z*- and *p*-values and CIs.

#### Eye-tracking analysis

Here we tested the hypothesis that attention mediates the relationship between hunger state and choice across domains (H2). Specifically, we predicted that hungry participants are more likely to look at tasty options which in turn increases the probability of tasty choices (H2a). Again, we predicted a spillover effect on intertemporal and social decisions. That is, hungry participants are more likely to look at impatient and selfish options which in turn increases the probability of impatient (H2b) and selfish decisions (H2c).

A prerequisite to test the mediating effect of attention on choice across domains (H2) was the effect of hunger state on choice. As this was only found in the food domain, we did not perform mediation analyses for the other two choice domains. For the food choice task we used a Bayesian within-subject multilevel mediation analysis^53^ using the bmlm R package^54^. We implemented a binary outcome (tasty vs healthy choice) and added hunger state as independent variable and relative DT proportion on tasty versus healthy options as mediating variable. In non-food tasks we assessed whether DT on hot attributes differed between conditions. We report correlation coefficients, standard errors (*SE*), as well as *z*- and *p*-values, CIs, as well as t-statistics.

#### Computational modelling analysis

Here we tested the hypothesis that the mechanisms through which hunger state affects choice across domains are the same, by comparing the effect of hunger state on the parameters in our cognitive model. We predicted that hunger state affects parameters irrespective of choice domain (H3).

We deviated from our preregistration to extend the multi-attribute time-dependent DDM with attentional parameters and instead used a variant of the recently proposed multi-attribute attentional DDM (maaDDM)^55^ to test how behavioral and attentional effects map onto underlying cognitive processes and thereby explain hunger-driven effects on choice and RT (for a detailed explanation please see . The maaDDM accounts for different impacts of the option’s underlying attributes as well as attentional dynamics with respect to option and attribute currently attended to. Here we use an extension of this model that we recently developed and that allows for attribute-specific attentional effects by assuming two $\phi$ parameters for the two attributes (maaDDM2 $\phi$)^2^. The maaDDM2 $\phi$ includes eight parameters: first, the boundary separation ($\alpha$), which determines the distance between the thresholds which when reached terminates the choice process. Second, a relative starting point bias ($\beta$), which refers to a systematic shift to either decision boundary and reflects preferences or expectations that influence the choice prior to evidence accumulation Third, the non-decision time (ndt) which reflects processes unrelated to the choice process. Fourth, a scaling parameter ($\delta$) which multiplied with the value difference (*VD*) determined the drift rate. *VD* was determined by the attribute-specific *VD* of the options *i* and *j* (i.e., *i* = hot option—taste, delay and self—and *j* = cold option—health, amount and NGO), weighted by the fifth free parameter $\omega$ (relative weight of hot attribute) and (1-$\omega$) (relative weight of cold attribute). Moreover, the model included the relative DT on each of the options’ underlying attributes (${f}_{i, A1}$, ${f}_{i, A2}$, ${f}_{j, A1}$, ${f}_{j, A2}$), where A1 and A2 refer to hot and cold attributes, respectively. Depending on the participants fixation, the non-looked at option was discounted with the sixth free parameter $\theta$ and the non-looked at attribute was discounted with the last two free parameters ${\phi }_{A1}$ and ${\phi }_{A2}$, for the hot and cold attribute, respectively. Thus, the VD of the maaDDM2 $\phi$ is defined as follows:

$$\begin{aligned} VD = & f_{{i,A1}} (\omega (A1_{i} - \theta A1_{j} ) + (1 - \omega )\phi _{{A2}} (A2_{i} - \theta A2_{j} )) \\ & + f_{{j,A1}} (\omega (\theta A1_{i} - A1_{j} ) + (1 - \omega )\phi _{{A2}} (\theta A2_{i} - A2_{j} )) \\ & + f_{{i,A2}} (\omega \phi _{{A1}} (A1_{i} - \theta A1_{j} ) + (1 - \omega )(A2_{i} - \theta A2_{j} )) \\ & + f_{{j,A2}} (\omega \phi _{{A1}} (\theta A1_{i} - A1_{j} ) + (1 - \omega )(\theta A2_{i} - A2_{j} )) \\ \end{aligned}$$

We implemented a hierarchical Bayesian parameter estimation^56^ in JAGS^57^. Specifications on the parameter estimation and model fit are documented in Table S15, and parameter recoveries are shown in Figs. S10–S12. In the food and social choice tasks, all individual parameters converged as assessed by the Gelman-Rubin Statistics (“Rhat”) with a threshold of 1.05^58^. In the intertemporal choice task one participant had elevated Rhats across parameters (Rhat < 2.5), but group-level parameters remained below the threshold. While exclusion of this participant led to successful convergence, it did not change posterior distributions. Consequently, and for better comparability between tasks, all modelling analyses included all 70 participants.

## Results

There was no difference in hunger ratings between conditions at lab arrival (${t}_{t0}$(125.22) = − 1.393, *p* = 0.166, *d* = − 0.244). In the sated condition hunger ratings were lower after the consumption of the protein shake (${t}_{t0 vs t1 (sated)}$(64) = − 11.439, *p* < 0.001, *d* = − 1.43). Whereas hunger ratings increased throughout the session in the hungry condition (${t}_{t0 vs t3 (hungry)}$(64) = 8.885, *p* < 0.001, *d* = 1.111). Finally, we also assessed whether hunger ratings differed between conditions at the timepoint before participants started the choice task (in sated after the consumption after the protein shake and in hungry before the choice tasks). We found that hunger ratings were significantly lower in the sated compared to the hungry condition (${t}_{t1 sated vs t2 hungry}$(112.24) = − 16.324, *p* < 0.001, *d* = − 1.541) (Fig. S1a).

### Domain-specific effect of hunger state on choice and RT

**Fig. 2:**
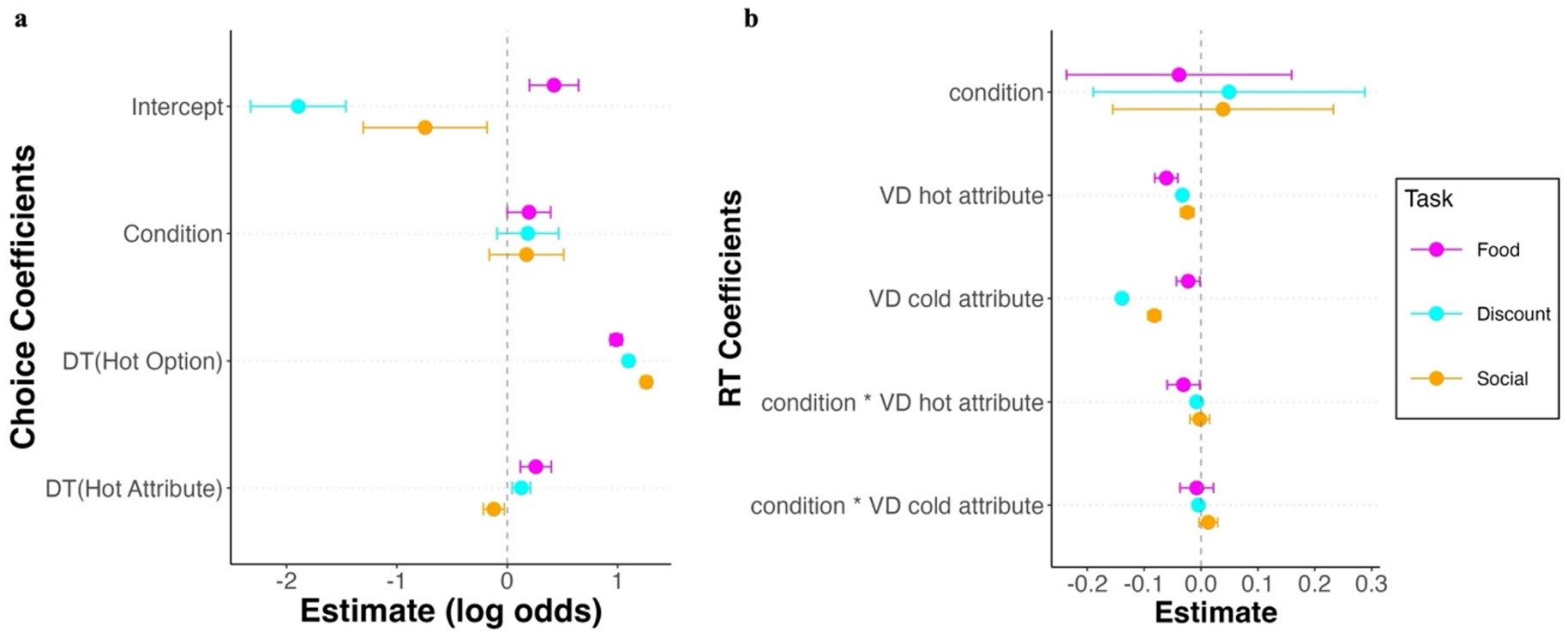
Predictors of choice and RT across domains. (**a**) Coefficients from the choice GLMMs across task; points represent group-level coefficients (log odds) with 95% CIs; Choice was coded as 1 = hot option and 0 = cold option across tasks; Coefficients of which CIs do not cross the dashed vertical line at 0 increase/ decrease the probability of choice. (**b**) Coefficients from the RT GLMMs across task; intercept not shown for visualization purposes of the predictors of interest; points represent group-level coefficients with 95% CIs; RT was in seconds; coefficients of which the CIs do not cross the dashed vertical line at 0 increase/ decrease RT.

In the food choice task, participants were more likely to select tasty options (${\beta }_{intercept}$=0.424, *SE* = 0.113, *z* = 3.744, *p* < 0.001). In the other two tasks, participants generally preferred patient (${\beta }_{intercept}$=-1.892, *SE* = 0.22, *z* = − 8.596, *p* < 0.001) and prosocial (${\beta }_{intercept}$= − 0.743, *SE* = 0.286, *z* = − 2.599, *p* = 0.009) options (Fig. 2a, Tables S1, S5, S9). This preference was also reflected in the effect of the attribute values on choice: values for taste, amount and NGO had a stronger impact on choice than values for health, delay and self (Fig. S2). In line with our hypothesis, hungry participants were more likely to select tasty food options than sated ones (${\beta }_{condition}$=0.198, *SE* = 0.099, *z* = − 1.986, *p* = 0.049). Against our hypothesis, we did not observe an effect of hunger state on intertemporal discounting (${\beta }_{condition}$=0.188, *SE* = 0.144, *z* = − 1.305, *p* = 0.192) and social choice (${\beta }_{condition}$=0.175, *SE* = 0.172, *z* = 1.018, *p* = 0.309) (Fig. 2a, Tables S1, S5, S9). Hungry participants who made more tasty decisions in the food choice task were also more likely to be choose less patiently in the intertemporal discounting task but did not choose differently in the social choice task (Fig. S3). A supplementary analysis in which we used the change in hunger rating as predictor of choice in addition to our attentional predictors demonstrated even stronger domain-specific hunger state effects on choice thereby providing converging evidence (Tables S2, S6, S10).

The RTs across tasks were unaffected by hunger state (food: ${\beta }_{condition}$= − 0.038, *SE* = 0.101, *z* = − 0.38, *p* = 0.704; intertemporal discounting: ${\beta }_{condition}$=0.05, *SE* = 0.122, *z* = 0.409, *p* = 0.684; social: ${\beta }_{condition}$=0.039, *SE* = 0.099, *z* = 0.393, *p* = 0.694) (Fig. 2b, Tables S4, S8 and S12). Across tasks a larger value difference of the hot and cold attributes decreased RT (food: ${\beta }_{taste VD}$= − 0.061, *SE* = 0.101, *z* = − 5.89, *p* < 0.001; ${\beta }_{health VD}$= − 0.022, *SE* = 0.011, *z* = − 2.213, *p* = 0.033 intertemporal discounting: ${\beta }_{delay VD}$=0.033 *SE* = 0.001, *z* = − 23.505, *p* < 0.001; ${\beta }_{amount VD}$=0.139, *SE* = 0.001, *z* = 106.004, *p* < 0.001; social: ${\beta }_{self VD}$=0.024, *SE* = 0.006, *z* = − 3.976, *p* < 0.001; ${\beta }_{NGO vd}$=0.082, *SE* = 0.006, *z* = − 14.372, *p* < 0.001), indicating that easier trials (i.e., larger absolute value difference) were faster irrespective of attribute type (Fig. S2). In the food domain, hunger state amplified the effect of decision difficulty on RT for larger taste VDs (${\beta }_{condition * taste VD}$=-0.031, *SE* = 0.015, *z* = − 2.101, *p* = 0.036), but not for smaller health VDs (${\beta }_{condition *health VD}$=-0.008, *SE* = 0.015, *z* = − 0.493, *p* = 0.622). In the intertemporal domain, hunger state amplified the effect for both the delay (${\beta }_{condition * delay VD}$= − 0.007, *SE* = 0.001, *z* = − 5.112, *p* < 0.001) and the amount attribute (${\beta }_{condition * amount VD}$= − 0.004, *SE* = 0.001, *z* = − 3.117, *p* = 0.002). In the social domain, we found neither an interaction effect between hunger state and self VD (${\beta }_{condition * self VD}$=-0.002, *SE* = 0.008, *z* = − 0.243, *p* = 0.808), nor between hunger state and NGO VD (${\beta }_{condition *NGO VD}$=0.013, *SE* = 0.008, *z* = 1.539, *p* = 0.124).

In sum, we found an effect of hunger state on choice within, but not across domains. Although hungry individuals were consistently more likely to select tasty over healthy options, we observed substantial interindividual variability in how hunger state affected choice across domain. Across tasks, there was no main effect of hunger state on decision speed.

### Domain-specific effects of hunger state on attention and choice

**Fig. 3:**
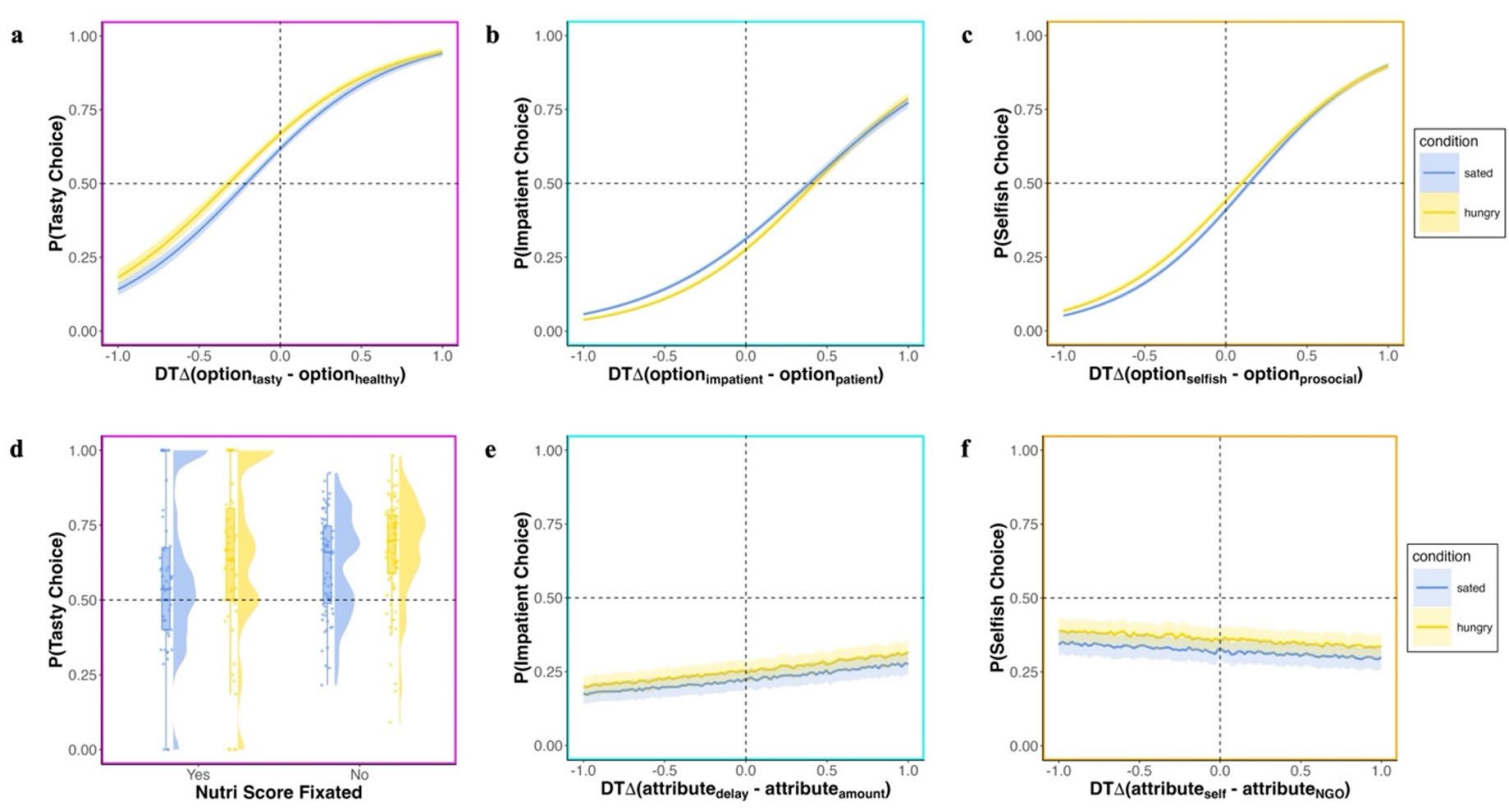
Effect of attention and hunger state on choice across domains. (**a**) Proportion of tasty choice given DT on ${\mathrm{option}}_{i}$(tasty) vs. ${\mathrm{option}}_{j}$(healthy); looking longer at the tasty option increases the proportion of tasty choice; the shift to the left compared to the black dashed lines indicates a general preference for choosing tasty options; the steeper slope in the hungry condition indicates a stronger effect of DT on the tasty option on choice (**b**) Proportion of impatient choice given DT on ${\mathrm{option}}_{i}$(impatient) vs ${\mathrm{option}}_{j}$ (patient); looking longer at the impatient option increases the proportion of impatient choice, the curve’s shift to the right indicates a preference for patient options; (**c**) Proportion of selfish choice given DT on ${\mathrm{option}}_{i}$(selfish) vs ${\mathrm{option}}_{j}$(prosocial); looking longer at the selfish option increases the proportion of selfish choice (**d**) Proportion of tasty choice by trials in which the Nutri-Score was fixated; fixating the Nutri-Score decreased the proportion of tasty choices (**e**) proportion of impatient choice as a function of DT difference of amount—delay attribute, looking longer at delay increased the proportion of impatient choice (**f**) Proportion of selfish choice as a function of DT difference of self – NGO attribute, no effect of looking longer at self attributes on selfish choice.

Across tasks, higher dwell time (DT) on one option was predictive of choosing this option: In the food choice task, looking longer at the tasty option predicted tasty choice (${\beta }_{DT(tasty)}$= 0.988, *SE* = 0.027, *z* = 36.89, *p* < 0.001) (Fig. 3a, Table S1); in the intertemporal discounting task looking longer at the impatient option predicted impatient choice (${\beta }_{DT(impatient)}$= 1.101, *SE* = 0.021, *z* = 51.715, *p* < 0.001) (Fig. 3b, Table S5) and in the social choice task looking longer at the selfish option predicted selfish choice (${\beta }_{DT(selfish)}$= 1.261, *SE* = 0.022, *z* = 56.921, *p* < 0.001) (Fig. 3c, Table S9). As the effect of hunger state on choice was domain-specific, the examination of the mediating role of attention in this regard was confined to the food domain. This analysis revealed that attention mediated the effect of hunger state on food choice. Specifically, hungry individuals were more likely to spend more time on tasty options (*M*_*a*_ = 0.056, *SE*_*a*_ = 0.03, *CI*_*a*_ = [0.003, 0.11]), which in turn predicted tasty choice (*M*_*b*_ = 1.1, *SE*_*b*_ = 0.08, *CI*_*b*_ = [0.94, 1.27]). Importantly, the direct path between hunger state and choice (*M*_*c*_ = 0.27, *SE*_*C*_ = 0.12, *CI*_*c*_ = [0.03, 0.52]) was no longer significant when attention was considered, indicating that attention fully mediated the relationship between hunger state and choice (*M*_*cp*_ = 0.19, *SE*_*cp*_ = 0.11*, CI*_*cp*_ = [− 0.024, 0.4]) (Table S13, S14). Exploratory analyses of the effects of hunger state on attention allocation in the non-food domains revealed no differences between conditions. Specifically, hungry participants were neither more likely to look at impatient options in the intertemporal discounting task (${t}_{DT(impatient option)}$(69) = 0.68, *p* = 0.499, *d* = 0.082) nor at selfish options in the social choice task ${t}_{DT(selfish option)}$(69) = 1.169, *p* = 0.247, *d* = 0.141).

The relationship between relative DT on the preferred attribute and choice was positive in the food and intertemporal choice domain. In the food choice task, not fixating the Nutri-Score increased the proportion of tasty choice (${\beta }_{DT(nutri)}$=0.26, *SE* = 0.072, *z* = 3.623, *p* < 0.001) (Fig. 3d, Table S1). In the intertemporal discounting task, looking longer at the delay attribute increased probability of impatient choice (${\beta }_{DT(delay)}$=0.129, *SE* = 0.043, *z* = 3.026, *p* = 0.002) (Fig. 3e, Table S5), but there was considerable variability between participants (Fig. S4). In the social choice task, selfish choice was overall negatively affected by the relative DT difference on the attribute for self versus NGO (${\beta }_{DT(self)}$= − 0.121, *SE* = 0.048, *z* = 2.51, *p* = 0.012) (Fig. 3c, Table S9). Again, examination of the individual slopes revealed that for some participants the effect of attribute attention on choice was positive, whereas it was negative or absent in others (Fig. S5). In addition, DT on hot attributes differed between conditions only in the food choice task (${t}_{DT(taste)}$(69) = 2.595, *p* = 0.012, *d* = 0.312; ${t}_{DT(delay)}$(69) = -0.846, *p* = 0.4, *d* = − 0.102; ${t}_{DT(self)}$(69) = 0.395, *p* = 0.694, *d* = 0.048) (Fig. S7). First and last fixations, as well as transition patterns are shown in Fig. S8.

In sum, attention allocation predicted choice across domains, but differences between conditions in attentional allocation were domain-specific. Thus, hungry participants were more likely to look at tasty options and disregarded Nutri-Scores, which lead to a higher proportion of tasty decisions. In the non-food domains, participants’ attentional allocation patterns remained unaffected by hunger state.

### Cognitive mechanisms underlying the effect of attention and hunger state on choice

**Fig. 4:**
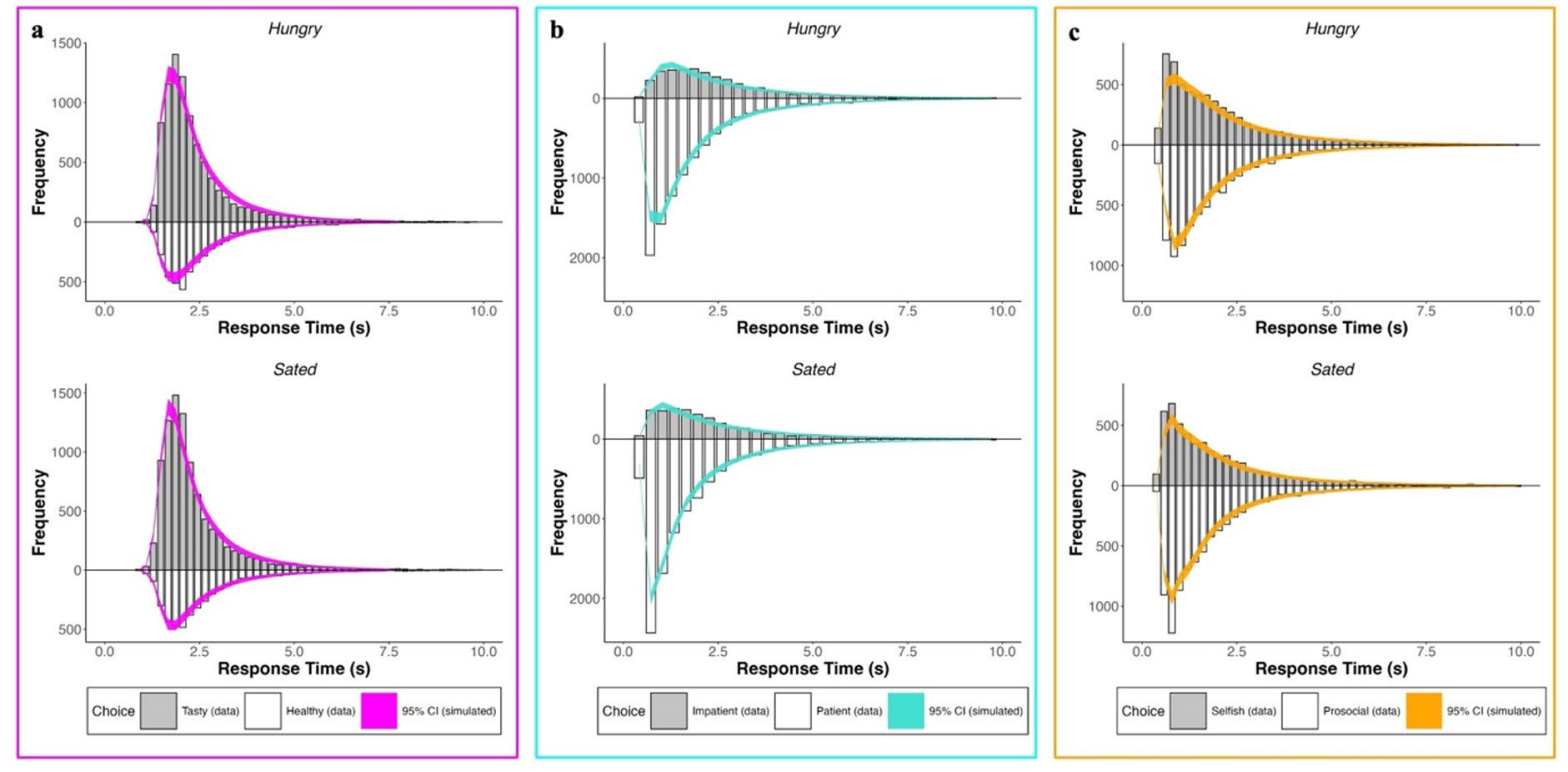
Posterior predictive checks across tasks. We simulated data by drawing 1000 parameter values from the posterior parameter distributions of each participant (**a**) RT distributions of the food choice task by choice (grey = tasty, white = healthy) with 95% credibility interval (CI) from our simulated data (pink) (**b**) RT distributions of the intertemporal discounting task by choice (grey = impatient, white = patient) with 95% credibility interval (CI) from our simulated data (turquoise) (**c**) RT distributions of the social choice task by choice (grey = selfish, white = prosocial) with 95% credibility interval (CI) from our simulated data (orange); respectively for hungry (upper row) and sated condition (lower row).

**Fig. 5:**
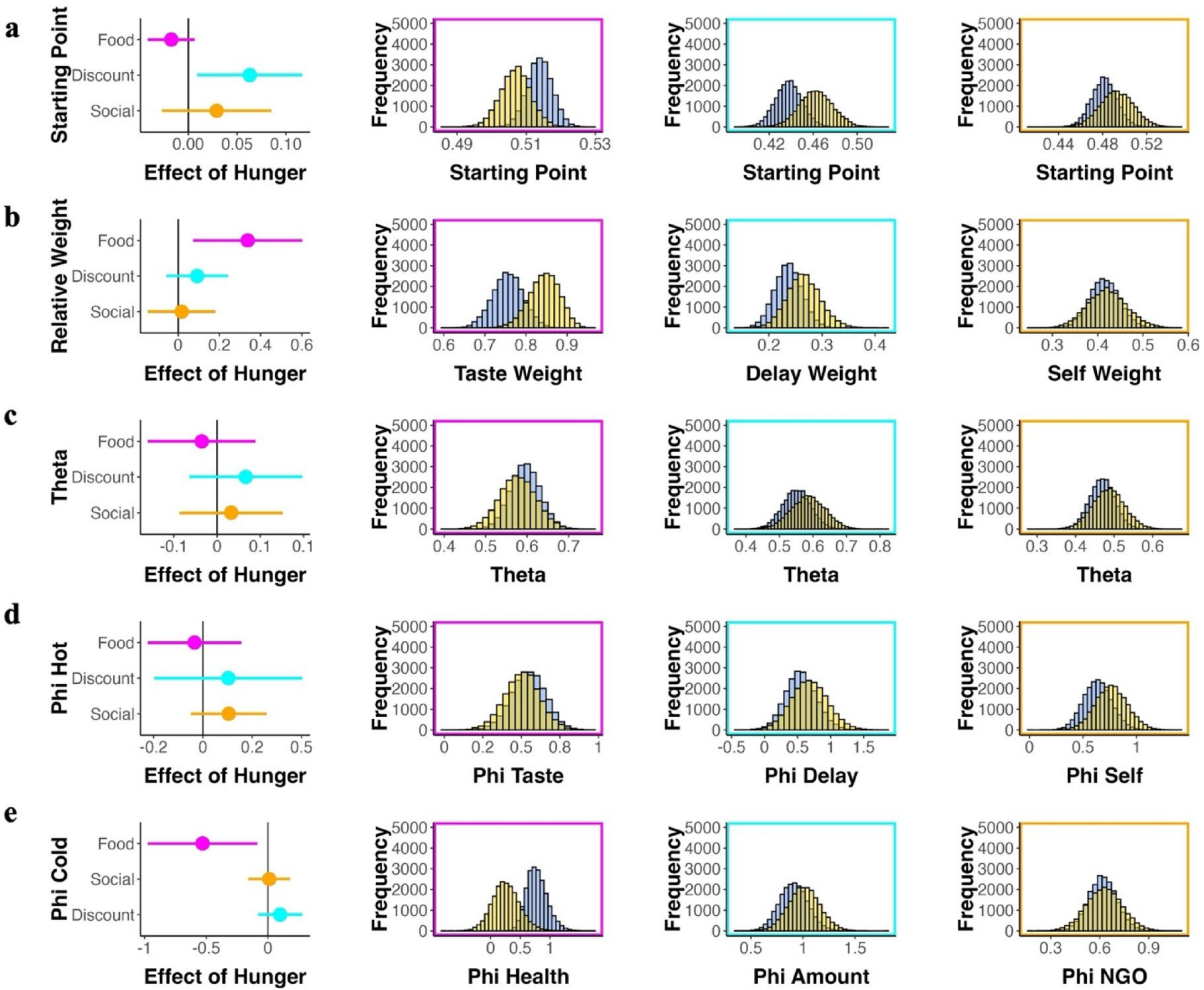
Selected parameter estimates of the maaDDM2 $\upphi$ across choice tasks*.* Effect of hunger state on group-level parameter estimates of the maaDDM2 $\upphi$ across tasks (pink = food; turquoise = intertemporal discounting; orange = social) on the left and the distributions per condition (blue = sated; yellow = hungry) on the right (**a**) no effect of hunger state on starting point bias in food and social choice task, in the intertemporal discounting task participants are biased toward later larger rewards, but less strongly under hunger (**b**) hunger state increases the relative taste weight, but does not impact the relative weight on delay and self; (**c**)**–**(**d**) no effect of hunger state on $\theta$ and ${\phi }_{hot}$ (**e**) hunger state increases attentional discounting of health, but does not impact attentional discounting of ${\phi }_{amount}$ and ${\phi }_{ngo}$.

We applied the maaDDM2 $\phi$, to test how hunger state affected the underlying cognitive mechanisms across choice tasks. Our model provided a very good fit of the data across all three choice tasks, evidenced by the posterior predictive checks, which yielded a very close match between predicted and actual choice and RT data (Fig. 4). To test for effects of hunger on decison-making processes we focus on our parameters of interest: $\beta$, $\omega$, $\theta$, as well as ${\phi }_{hot}$ and ${\phi }_{cold}$ (see Fig. S9 for the posterior distributions of the remaining parameters). To this end, we examine the parameter’s highest density intervals (HDI), which reflect 95% of values in the posterior distribution, for each condition (Fig. 5). As a robustness check, we fitted a Bayesian standard hyperbolic discounting model^59,60^ for the intertemporal choice task (Fig. S10), which yielded converging evidence for domain-specific effects of hunger state on choice.

The starting point was not affected by hunger state in the food ($HDI_{{\beta \left( {Food} \right)}}$= [− 0.041, 0.007]) and the social choice task ($HDI_{{\beta \left( {Social} \right)}}$= [− 0.027, 0.085]), but in the intertemporal discounting task (Fig. 5a): Participants were generally biased towards patient decisions in both conditions as the HDIs did not include 0.5 ($HDI_{{\beta \left( {Discount sated} \right)}}$= [0.414, 0.462]; $HDI_{{\beta \left( {Discount hungry} \right)}}$= [0.432, 0.493]). However, under hunger participants were biased less strongly towards patient choice ($HDI_{{\beta \left( {Discount} \right)}}$= [0.009, 0.117]).

In line with participants’ general preference for tasty options, the HDI of the taste weight was larger than 0.5 in both conditions ($HDI_{{\omega \left( {Food, sated} \right)}}$= [0.698, 0.831]; $HDI_{{\omega \left( {Food, hungry} \right)}}$= [0.788, 0.922]). Similarly, the higher proportion of patient choice mapped onto a relatively smaller weight on the attribute for delay in both conditions ($HDI_{{\omega \left( {Discount, sated} \right)}}$= [0.144, 0.256]; $HDI_{{\omega \left( {Soical, hungry} \right)}}$= [0.174, 0.324]) and the higher proportion of prosocial decisions in both conditions mapped onto a relatively smaller weight on the attribute for self in both conditions ($HDI_{{\omega \left( {sated} \right)}}$= [0.324, 0.48]; $HDI_{{\omega \left( {hungry} \right)}}$= [0.321, 0.53]). Importantly, the difference in tasty choice between conditions in the food choice task mapped onto significant difference in relative taste weight between conditions ($HDI_{{\omega \left( {Food} \right)}}$= [0.112, 0.642]) (Fig. 5b). In the non-food tasks, we did not find differences in relative weights between conditions ($HDI_{{\omega \left( {Discount} \right)}}$. =[− 0.032, 0.099]; $HDI_{{\omega \left( {Social} \right)}}$=[− 0.146, 0.18]).

Across tasks, there were no differences between conditions in discounting the non-looked at option across tasks ($HDI_{{\theta \left( {Food} \right)}}$= [− 0.092, 0.017], $HDI_{{\theta \left( {Discount} \right)}}$= [− 0.032, 0.099], $HDI_{{\theta \left( {Social} \right)}}$= [− 0.043, 0.076]) (Fig. 5c). In the food choice task, the HDIs of $\theta$ were relatively high in both conditions ($HDI_{{\theta \left( {Food, sated} \right)}}$= [0.619, 0.715]; $HDI_{{\theta \left( {Food, hungry} \right)}}$= [0.565, 0.714]) as compared to the HDI distribution of the $\theta$ in the non-food tasks in both conditions ($HDI_{{\theta \left( {Discount sated} \right)}}$= [0.468, 0.634]; $HDI_{{\theta \left( {Discount hungry} \right)}}$= [0.484, 0.683]; ($HDI_{{\theta \left( {Social sated} \right)}}$= [0.422, 0.546]; $HDI_{{\theta \left( {Social hungry} \right)}}$= [0.417, 0.579]). This indicated that the non-looked-at option was discounted more in the non-food compared to the food tasks.

Across tasks, there are no differences in discounting the non-looked at hot attribute ($HDI_{{\phi \left( {taste} \right)}}$= [− 0.291, 0.16]; $HDI_{{\phi \left( {delay} \right)}}$= [− 0.079, 0.278]; $HDI_{{\phi \left( {self} \right)}}$= [− 0.06, 0.323]) (Fig. 5d). Overall, HDI distribution of the $\phi_{hot}$ was comparable across tasks and conditions ($HDI_{{\phi \left( {taste; sated} \right)}}$= [0.29, 0.725]; $HDI_{{\phi \left( {taste; hungry} \right)}}$= [0.211, 0.669]; $HDI_{{\phi \left( {delay; sated} \right)}}$= [0.106, 1.042]; $HDI_{{\phi \left( {delay; hungry} \right)}}$= [0.15, 1.238]; $HDI_{{\phi \left( {self; sated} \right)}}$= [0.381, 0.901]; $HDI_{{\phi \left( {self; hungry} \right)}}$= [0.473, 1.065]). This indicates that the unattended hot attribute was similarly discounted across tasks and conditions. In the intertemporal discounting task, the HDI of $\phi_{delay}$ almost covers the entire range between 0 and 1, indicating potential individual differences in how the delay attribute affects evidence accumulation if not looked at.

In addition to the difference in relative weighting between conditions in the food choice task, our modeling results suggest that hunger state affected the discounting of the non-looked at cold attribute (${HDI}_{\phi (health)}$= [− 1.088, − 0.188]) (Fig. 5e). This indicates that hungry participants do not consider health when not overtly attending to it (${HDI}_{\phi (health; hungry)}$= [− 0.352, 0.611]), while sated participants do (${HDI}_{\phi (health; sated)}$= [0.434, 1.119]). In the non-food tasks, we did not find an effect of hunger state on ${\phi }_{cold}$ (${HDI}_{\phi (amount)}$= [− 0.079, 0.278]; ${HDI}_{\phi (ngo)}$= [− 0.158, 0.18]). In the intertemporal discounting task, the HDI being centered around 1 in both conditions (${HDI}_{\phi (amount; sated)}$= [0.69, 1.23]; ${HDI}_{\phi (amount; hungry)}$= [0.761, 1.366], demonstrates that even when participants do not look at the monetary amount, it strongly influences choice indicating that money can easily be kept in working memory.

In sum, the maaDDM2 $\phi$ provides additional evidence for domain-specific effects of hunger state on choice. While hunger state alters the choice process in the food choice task by increasing both the relative importance of taste and the attentional discounting of health information, the parameters remained largely unaffected by hunger state in the intertemporal and social choice tasks.

## Discussion

The aim of this study was to shed light on the effects of hunger state within and across domains. We provide threefold evidence for the conclusion that hunger state affects choices within but not across domains. First, on the behavioral level, we show that hunger state leads to less healthy food choice but does not affect social and intertemporal choice. Second, we show that while visual attention predicts choice across domains, it is affected by hunger state only in the food domain, where it amplifies its effect on choice. Third, our modeling analyses reveals that hunger state affects relative weighting and attribute specific attentional discounting in the food domain but not in other choice domains. Together our findings suggest that hunger state does not lead to general changes in the decision-process but rather domain-specific alterations.

Notably, the literature on the effects of hunger state on choice in non-food domains is mixed. On the one hand, studies indicate that hungry individuals make more selfish^12–14^, risky^61^ and less moral^62^ and patient^11,63,64^ decisions. On the hand, other studies find opposing effects, for example hungry individuals discount later larger rewards less strongly^65^ and express more support for social welfare, despite not acting upon it^66^. And yet other studies^15,67–71^ do not report effects of hunger state on choice in non-food domains. Our results are most in line with the latter. One reason for these mixed findings may be the hunger state manipulation. It has been shown that different macro-nutritional ratios lead to differences in decision-making. For example, the consumption of a meal with a high compared to low carbohydrate-to-protein ratio increased participants’ social punishment behavior in response to norm violations^72^. This fits well with a study^62^ that demonstrated increases in moral disapproval of ethical violations after consuming bananas, compared to consuming nothing. Whereas the primary function of carbohydrates is supplying energy via glucose in the brain, the protein metabolism creates new structural and functional components with energy supply as a secondary function^73,74^. Our protein manipulation reduced hunger ratings, which is in line with a recent meta-analysis^75^, and protein consumption has been linked to reductions in reward driven eating behavior^76^. The domain-specific finding in our study could consequently indicate that protein’s satiation signals are biologically specific to the food reward circuitry. However, we cannot rule out that our incentive structure in the non-food domains (i.e., book coupons and NGO donations), might not have been salient enough to reveal hunger effects domains. To address these competing explanations, future research may consider implementing different macro-nutritional manipulations to elicit satiety and consider keeping the incentive commodity constant between tasks (i.e., food or money).

In line with previous work^18^, we demonstrate a strong relation between gaze and choice across different value-based choice contexts. We show that beyond option-based attentional effects on choice, the attention to the attributes also play an important role in the choice process across different choice contexts, which aligns with recent work in the field^55^. Our findings highlight that the extent to which attention influences choice can be state dependent. This connection has also been suggested in a recent study^77^ in which the authors showed that a positive mood induction augmented the attentional direction bias toward costly choice options. Here, we refine this link by showing that state dependent attentional shifts occur exclusively in domains where the state is relevant to the choice (i.e., domain-specific). Specifically, we demonstrate domain-specific attenuation of attention under hunger, that is hungry participants were more likely to look at tasty options which in turn increased probability of choice, but hunger state did not affect attention or choice in the other two domains. Our interpretation of state dependent attentional shifts being domain-specific is also supported by other work^78^, which did not find an effect of hunger state on attention in non-food tasks. Specifically, the results of the study suggest that hungry versus sated participants neither demonstrate lower attentional control in a Go/No-Go task, nor search for information differently in an information gathering task. Critically, our findings extend those found in the perceptual attention task^78^ to preferential decision-making, suggesting consistency in the domain-specific influence of hunger state across lower-level perceptual and higher-level value-based decisions.

In line with the behavioral and attentional results, our modeling results suggest that the choice processes in hungry and sated states differ only in food but not across non-food choice domains. In this, the modelling results complement the descriptive behavioral analyses explaining the mechanisms underlying hunger state on choice across domains. The parameters determining the drift rate are affected by hunger state only in the food, but not in the intertemporal and social domain. Specifically, we find that the higher proportion of tasty decisions under hunger is due to increased importance of the taste attribute (i.e., $\omega$) and increased attentional discounting of the health attribute (i.e., ${\phi }_{cold}$), rather than a shift in pre-decisional bias (i.e., starting point). One interesting and unanticipated result worth pointing out is the effect of hunger state on the starting point parameter in the intertemporal discounting task. While participants were generally biased toward later larger rewards, this bias was less pronounced under hunger. The starting point bias contributes to explaining the extreme preference for later larger rewards in general and might potentially be a result of both our design (i.e., random pairs of sooner smaller versus later larger rewards) and incentivization scheme (i.e., book vouchers were worth the wait). The attenuation of the starting point bias in the intertemporal discounting task under hunger fits well with a recent study reporting a shift in starting point bias towards sooner smaller rewards using erotic cues^79^, indicating that intertemporal choices are sensitive to internal states. Importantly, the hunger-induced shift in bias in the intertemporal discounting task differs substantially to that found in the food choice task, according to which hunger state affects the choice process itself by increasing the importance assigned to the relative taste weight and increasing the attentional discounting of the health information. Many intertemporal choice models do not assume an attribute-wise choice process but rather that delay and amount of an option are integrated (for common models see ref^80^.). However, others^35,81,82^ provided evidence for an attribute-wise comparison process, which we have also successfully adopted here. While this model allowed us to make comparisons with other tasks, we recommend additional studies with an adaptive intertemporal choice design and careful model comparison to reveal how hunger state impacts intertemporal discounting.

A few limitations of the present study are worth pointing out. Even though we successfully manipulated hunger state, by carefully considering participants age, gender and weight, the short-term effects of protein on the reward system are less well understood compared to other macronutrients. We speculate that the implementation of carbohydrate manipulation might have yielded stronger effects of hunger state on choice potentially spilling over across non-food domains. Thoroughly disentangling the biological mechanisms of macronutrients on decision-making by implementing controlled cross-over studies with different nutritional manipulations will shed light onto divergent dietary effects on choice across domains. Another limitation may be the different visualizations of the attributes between tasks. While we used squares for the intertemporal and the social choice task, we implemented food stimuli and their corresponding nutritional scores. These different visual representations induced markedly different attention patterns between tasks (Fig. S7). However, by controlling for attention in our GLMMs we mitigate the effects of the visually different stimuli between task on choice. In addition, we have opted for an ecological representation in the food choice task which has been commonly used in the field. Comparable to our results, a study^83^ found a high proportion of dwell time on food images compared to nutritional scores. Importantly, the authors show that, if participants were given nutritional scores compared to when they only saw food images, the proportion of healthy decisions increased, indicating the effectiveness of the health attribute visualization. Nevertheless, future studies should aim to improve comparability of stimuli representations between tasks. For example, one study^11^ used the same discounting stimuli within each of their three choice domains and found domain-general effects of hunger state on choice. Recently, a study^84^ demonstrated that a food choice task using words (e.g., 50% tasty and 100% healthy) rather than food images led to more healthy decision making, potentially due to lower choice conflict. It would be interesting to assess whether the effects of hunger state on food choice could be replicated for such abstract representation, or whether additional cues in the non-food task (e.g., picture of participant and logo of NGO in the social choice task) may yield domain-general effects of hunger state.

In sum, our behavioral, eye-tracking and modeling results provide converging evidence that hunger state affects decision-making in a domain-specific manner. In the food domain, hungry individuals were more likely to look at and consequently select tasty over healthy options. Critically, we did not observe any hunger-driven attentional and choice shifts in non-food domains. That is, hunger neither increased impulsive nor selfish decision-making processes. Overall, our findings add to the current debate on whether and how hunger state affects choice, with important societal and economic implications, suggesting that the cognitive mechanisms underlying decision-making under hunger are confined to the food domain.

## Supplementary Information

Below is the link to the electronic supplementary material.

Supplementary Material 1

## Data Availability

The data that support the findings of this study are openly available on Zenodo (10.5281/zenodo.17284917).

## Code availability

All code is available on Github (https://github.com/JenniferMarch/Hunger_across_domains).
