# Supplementary material for: Domain-specific effects of hunger on attention and choice
Source: Sci Rep. 2026 Apr 21;16:13030. doi: 10.1038/s41598-026-48772-0 (PMC13100030; doi:10.1038/s41598-026-48772-0)
Supplement: Supplementary file 1 — Supplementary Material 1 [file 41598_2026_48772_MOESM1_ESM.pdf]

Supplements

Figure S1

Differences between ratings by condition

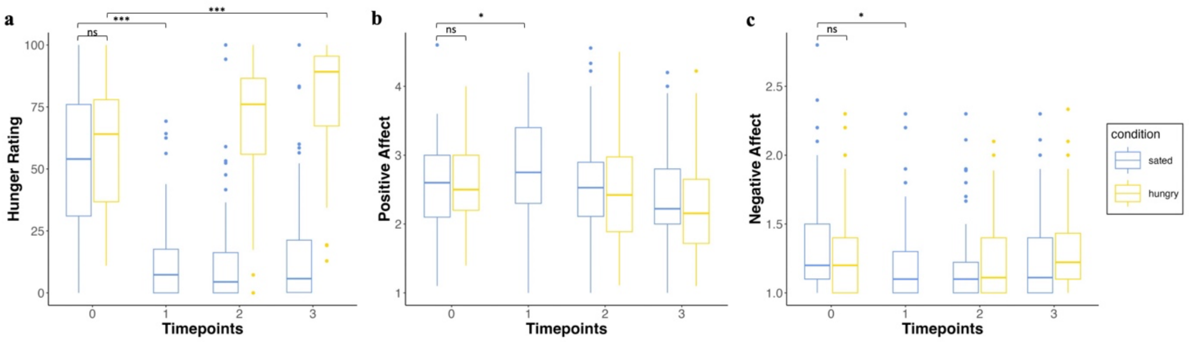

Note. **a)** there was no difference in hunger ratings between conditions at lab arrival (i.e., t_{t0}(125.22)=-1.393, *p*=.166, *d*=-0.244) and were decreased by the consumption of the protein shake ($t_{t0\ vs\ t1\ (sated)}$(64)=-11.439, *p*<.001, *d*=-1.43) in the sated condition and increased throughout the session in the hungry condition ($t_{t0\ vs\ t3\ (hungry)}$(64)=8.885, *p*<.001, *d*=1.111), given multiple tests we corrected alpha levels using Bonferroni Correction (α=.017); **b)** participants indicated no difference in positive affect at lab arrival, ($t_{PA\ t0}$(127.84)=-0.186, *p*=.853, *d*=-0.033), yet the protein shake elevated positive affect ($t_{PA\ t0\ vs\ t1\ (sated)}$(64)=2.605, *p*=.011, *d*=0.326) **c)** similarly, participants indicated no difference in negative affect at lab arrival, ($t_{NA\ t0}$(124.01)=0.474, *p*=.636, *d*=0.083), whereas their negative affect decreased after the consumption of the protein shake ($t_{NA\ t0\ vs\ t1\ (sated)}$(64)=2.543, *p*=.013, *d*=0.318); importantly, the effects of our manipulation on mood ratings are much smaller than the effects on hunger ratings.

Figure S2

Effects of attributes underlying choice across domains

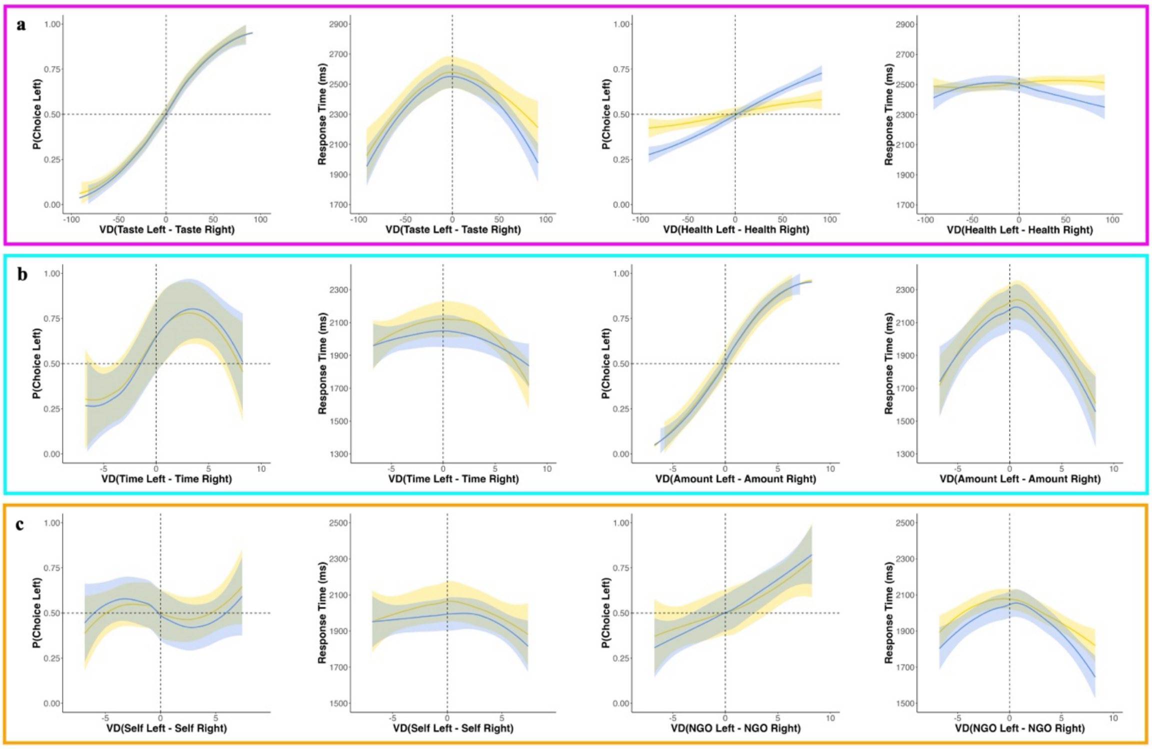

Note. **a)** From left to right: Probability to choose the left option as a function of taste VD (left-right) showing that tastier options were predictive of choice; Mean RTs as a function of taste VD, showing higher RTs for options of similar taste value; Probability to choose the left option as a function of health VD (left-right), showing that under hunger the dependency of choice on health information was marginal; mean RTs as a function of health VD, showing only a slight relation between health VD and RT (a)-e) adapted from March & Gluth, 2025) ; **b)** From left to right: Probability to choose the left option as a function of self VD (left-right); Mean RTs as a function of self VD, showing that under hunger the dependency of choice on value for self was marginal; Probability to choose the left option as a function of NGO VD (left-right), showing that options with a larger amount for the NGO were predictive of choice; mean RTs as a function of NGO VD, showing higher RTs for options with similar NGO values; **c)** Probability to choose the left option as a function of amount VD (left-right) showing that options with higher amounts were predictive of choice; Mean RTs as a function of amount VD, showing higher RTs for options of similar amounts; Probability to choose the left option as a function of time VD (left-right); mean RTs as a function of time VD, showing only a slight relation between time VD and RT; For illustration purposes, value differences were segmented into 25 bins, and a locally weighted scatterplot smoothing technique was applied with a span of 0.75. Transparent shades indicate the standard errors of the smoothed choice probability and RT for the respective value bins

Figure S3

Correlation of the effect of hunger state between tasks

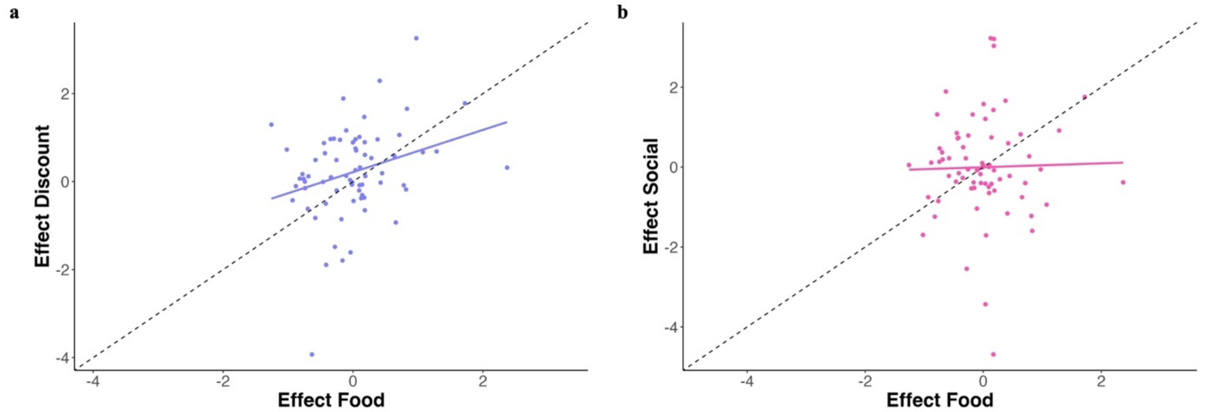

Note. **a)** correlation in effect of hunger state between food and intertemporal discounting task ($t(68)=2.555$, $p=0.013$, $d=0.31$), suggesting that hungry individuals which were more likely to select tasty options in the food choice task were also more likely to select impatient options in intertemporal choice task; **b)** no correlation in effect of hunger state between food and social choice task ($t(68)=0.196$, $p=0.845$, $d=0.024$). The dots represent the individual slopes for condition for each participant obtained by combining fixed and random effects of condition of the reported models for each task (**Table S1**; **Table S4**, **Table S7**).

Table S1

Effect of hunger state and attention on food choice

	Fixed Effects				
	coefficient	SE	z-statistic	p-value *(cor. p-value)*	CI
(Intercept)	0.424	0.113	3.744	<0.001	[0.202, 0.646]
condition (hungry)	-0.198	0.1	-1.970	0.049 *(0.146)*	[0.001, 0.395]

	Fixed Effects				
	coefficient	SE	z-statistic	p-value *(cor. p-value)*	CI
DT (tasty option)	0.998	0.027	36.317890	<0.001	[0.936, 1.041]
DT (nutri)	0.26	0.071	3.623	<0.001	[0.119, 0.401]

	Random Effects		
	Variance	SD	Correlation
(Intercept)	0.59	0.768	
condition (hungry)	0.531	0.729	-0.33

Note. Results of the GLMM tasty choice ~ condition + DT (tasty option) + DT (nutri) + (1+condition|subject), using binomial distribution with the logit link function and optimizing fit with bobyqa, 10458 observations in 70 participants, *AIC*=11165.4; DT (tasty option) referred to the relative scaled (on subject level) dwell time on the tasty versus healthy option; DT on the Nuti-Score was binarized, such that trials in which the Nutri-Score was fixated at all were coded with 0, while trials in which the Nutri-Score was not fixated were coded with 1; *SE*=standard error, we applied Bonferroni correction for multiple comparisons for corrected p-value in parentheses and italic; *CI*=confidence interval calculated using Wald approximation, *SD*=standard deviation.

Table S2

Effect of hunger rating on food choice

	Fixed Effects			
	coefficient	SE	z-statistic	p-value *(cor. p-value)*
(Intercept)	0.559	0.116	-4.816	<0.001
delta hunger	-0.004	0.002	-2.508	0.012 *(0.036)*
DT (tasty option)	0.988	0.027	36.309	<0.001
DT (nutri)	0.272	0.073	3.75	<0.001

	Random Effects		
	Variance	SD	
(Intercept)	0.617	0.786	
condition (hungry)	0.5	0.707	-0.31

Note. Results of the GLMM tasty choice ~ delta hunger + DT (tasty option) + DT (nutri) + (1 + condition | subject), using binomial distribution with the logit link function and optimizing fit with bobyqa, 10111 observations in 70 participants, *AIC*=10793.8; delta hunger, refers to the

difference in hunger ratings between two time points (sated: before protein shake – after protein shake administration; hungry: at lab arrival – before start of choice task); DT (tasty option) refers to the relative scaled dwell time on the tasty vs healthy option; DT (refers) refers to the relative scaled dwell time on the attribute for delay versus amount; given the large variation between participants with respect to the effect of DT on the Nuti-Score was binarized, such that trials in which the Nutri-Score was fixated were coded with 0, while trials in which the Nutri-Score was not fixated at all were coded with 1; *SE*=standard error, we applied Bonferroni correction for multiple comparisons for corrected p-value in parentheses and italic *SD*=standard deviation.

Table S3

Effect of hunger state, attention, mood and demographics on food choice

	Fixed Effects				
	coefficient	SE	z-statistic	p-value	CI
(Intercept)	0.24	0.638	0.376	0.707	[-1.011, 1.491]
condition (hungry)	0.179	0.203	0.884	0.377	[-0.218, 0.577]
DT (tasty option)	0.991	0.031	32.341	<0.001	[0.93, 1.051]
DT (nutri)	0.276	0.08	3.465	<0.001	[0.12, 0.433]
gender	-0.015	0.245	-0.061	0.952	[-0.495, 0.466]
BMI	0.01	0.023	0.422	0.672	[-0.035, 0.055]
age	-0.01	0.014	-0.734	0.463	[-0.038, 0.017]
Positive Affect	-0.14	0.112	-1.249	0.212	[-0.361, 0.08]
Negative Affect	-0.086	0.295	-0.29	0.772	[-0.665, 0.493]
Hunger Rating	0.001	0.003	0.263	0.792	[-0.005, 0.007]
EB (external)	0.111	0.217	0.512	0.609	[-0.315, 0.537]
EB (emotional)	-0.074	0.149	-0.496	0.62	[-0.366, 0.218]
EB (restrictive)	-0.017	0.145	-0.115	0.908	[-0.301, 0.267]

	Random Effects		
	Variance	SD	Correlation
(Intercept)	0.501	0.708	
condition (hungry)	0.517	0.719	-0.31

Note. Results of the GLMM tasty choice ~ condition + DT (tasty option) + DT (nutri) + gender + BMI + age + Positive Affect + Negative Affect + Hunger Rating + EB (external) + EB (emotional) + EB (restictive) + (1+condition|subject), using binomial distribution with the logit link function and optimizing fit with bobyqa, 7994 observations in 53 participants, *AIC*=8602.1; DT (tasty option) referred to the relative scaled (on subject level) dwell time on the tasty versus healthy option; DT on the Nuti-

Score was binarized, such that trials in which the Nutri-Score was fixated were coded with 0, while trials in which the Nutri-Score was not fixated at all were coded with 1; BMI and age were centralized; for Positive and Negative Affect as well as Hunger Rating we used the change score (t3-t0); EB refers to the eating behavior questionnaire; *SE*=standard error, *CI*=confidence interval calculated using Wald approximation, *SD*=standard deviation.

Table S4

Effect of hunger on RTs in food choice task

	**Fixed Effects**				
	coefficient	SE	z-statistic	p-value	CI
(Intercept)	2.666	0.103	25.995	<0.001	[2.465, 2.868]
condition (hungry)	-0.038	0.101	-0.38	0.704	[-0.236, 0.16]
taste VD	-0.061	0.01	-5.589	<0.001	[-0.081, -0.041]
health VD	-0.022	0.011	-2.13	0.033	[-0.043, -0.002]
condition x taste VD	-0.031	0.014	-2.101	0.036	[-0.059, -0.002]
condition x health VD	-0.007	0.015	-0.493	0.622	[-0.037, 0.022]

	**Random Effects**		
	Variance	SD	Correlation
(Intercept)	0.141	0.376	
condition (hungry)	0.165	0.407	-0.59
Residual	0.122	0.35	

Note. Results of the GLMM RT ~ condition * (tasteVD + healthVD) + (1+condition|subject), using a Gamma distribution with the identity link function and optimizing fit with bobyqa, 10458 observations in 70 participants, we used the absolute scaled taste and health value difference (VD); *AIC*=23332.5; *SE*=standard error, *CI*=confidence interval calculated using Wald approximation, *SD*=standard deviation.

Figure S4

Variation in the effect of relative attribute DT on impatient choice

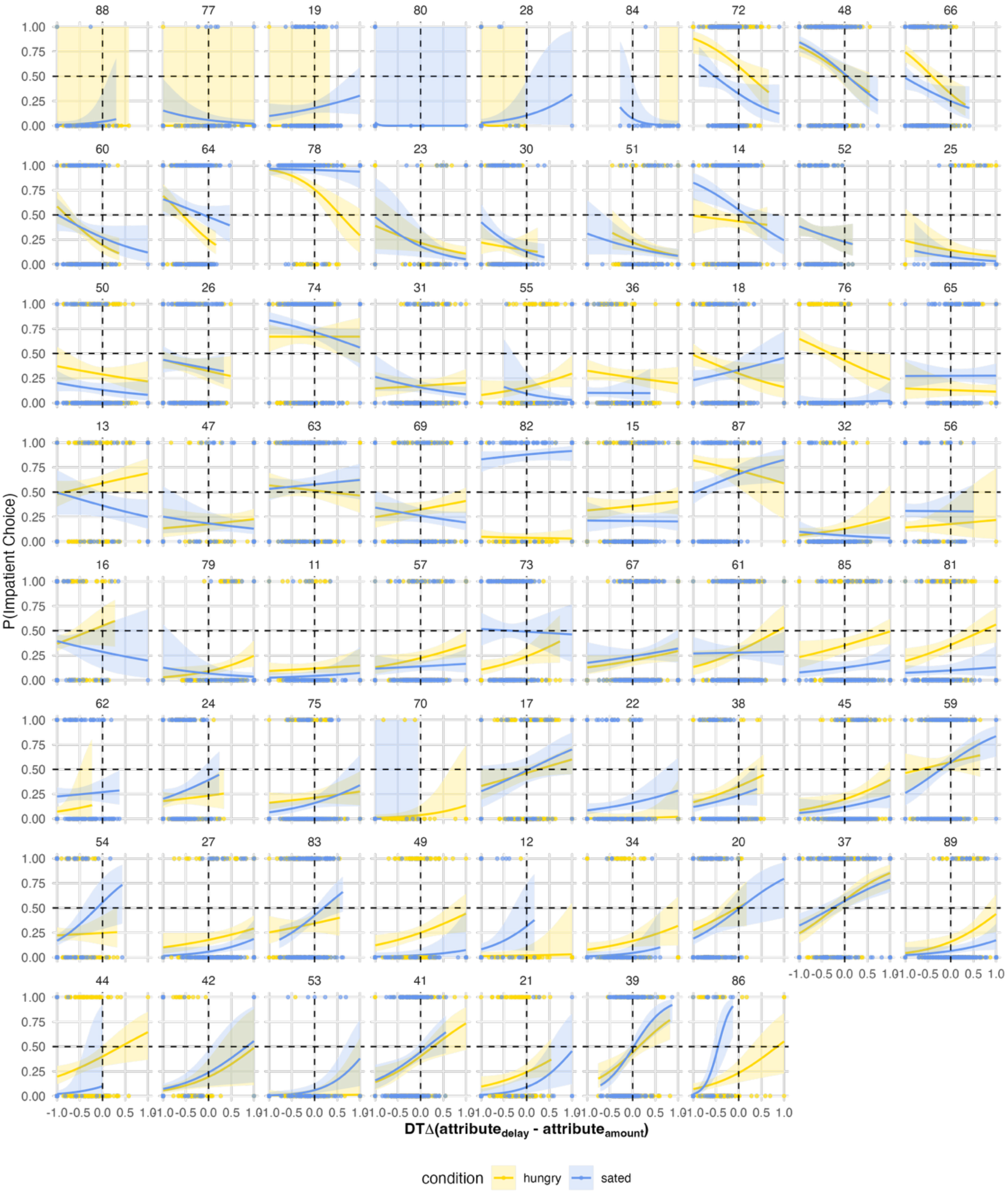

Note. Each subplot represents the effect of relative DT (delay – amount) on impatient choice of one participant, in each condition (yellow = hungry; blue = sated), in some participants looking longer at delay, decreased the probability of impatient choice (rows 1-2.5); in some participants looking longer at delay, did not affect probability of impatient choice (rows 2.5-4.5); in some participants looking longer at delay, increased the probability of impatient choice (rows 4.5-7).

Table S5

Effect of hunger on intertemporal discounting

	Fixed Effects				
	coefficient	SE	z-statistic	p-value *(cor. p-value)*	CI
(Intercept)	-1.892	0.22	-8.607	<0.001	[-2.323, -1.461]
condition (hungry)	0.188	0.142	1.319	0.187 *(0.532)*	[-0.091, 0.467]
DT (delay option)	1.101	0.021	51.838	<0.001	[1.059, 1.143]
DT (delay attribute)	0.129	0.042	3.068	0.002	[0.047, 0.212]

	Random Effects			
	Variance	SD	Correlation	
(Intercept)	3.274	1.809		
condition (hungry)	1.24	1.113	-0.37	
DT (delay attribute)	0.092	0.304	-0.39	0.01

Note. Results of the GLMM impatient choice ~ condition + DT (delay option) + DT (delay attribute) + (1 + condition + DT (delay attribute) | subject), using binomial distribution with the logit link function and optimizing fit with bobyqa, 24611 observations in 70 participants, *AIC*=19404.5; DT (delay option) refers to the relative scaled dwell time on the sooner smaller vs later larger option; DT (delay attribute) refers to the relative scaled dwell time on the attribute for delay versus amount; given the large variation between participants with respect to the effect of DT (delay attribute) (see **Figure S6**), we account for this using a random slope, which also improved AIC; *SE*=standard error, we applied Bonferroni correction for multiple comparisons for corrected p-value in parentheses and italic, *CI*=confidence interval calculated using Wald approximation, *SD*=standard deviation.

Table S6

Effect of hunger rating on intertemporal discounting

	Fixed Effects			
	coefficient	SE	z-statistic	p-value *(cor. p-value)*
(Intercept)	-1.775	0.206	-8.606	<0.001
delta hunger	-0.002	0.002	-0.688	0.491 *(1)*
DT (delay option)	1.105	0.022	51.421	<0.001
DT (delay attribute)	0.131	0.042	3.112	0.002

	Random Effects			
	Variance	SD	Correlation	
(Intercept)	3.436	1.854		
condition (hungry)	1.281	1.132	-0.4	
DT (delay attribute)	0.09	0.3	-0.4	-0.01

Note. Results of the GLMM impatient choice ~ delta hunger + DT (delay option) + DT (delay attribute) + (1 + condition + DT (delay attribute) | subject), using binomial distribution with the logit link function and optimizing fit with bobyqa, 23749 observations in 70 participants, *AIC*=18967.3; delta hunger, refers to the difference in hunger ratings between two time points (sated: before protein shake – after protein shake administration; hungry: at lab arrival – before start of choice task); DT (delay option) refers to the relative scaled dwell time on the sooner smaller vs later larger option; DT (delay attribute) refers to the relative scaled dwell time on the attribute for delay versus amount; given the large variation between participants with respect to the effect of DT (delay attribute), we account for this using a random slope, which also improved AIC; *SE*=standard error, we applied Bonferroni correction for multiple comparisons for corrected p-value in parentheses and italic *SD*=standard deviation.

Table S7

Effect of hunger state, attention, mood and demographics on intertemporal discounting

	Fixed Effects				
	coefficient	SE	z-statistic	p-value	CI
(Intercept)	-3.119	0.113	-2.802	0.005	[-5.301, -0.937]
condition (hungry)	0.344	0.255	1.349	0.177	[-0.156, 0.845]
DT (delay option)	1.166	0.025	47.51	<0.001	[1.118, 1.215]
DT (delay attribute)	0.135	0.048	2.811	0.005	[0.041, 0.229]
gender	-0.205	0.044	-0.465	0.647	[-1.067, 0.657]
BMI	-0.034	0.04	-0.854	0.393	[-0.113, 0.045]
age	-0.024	0.024	-1.004	0.315	[-0.072, 0.023]
Positive Affect	-0.059	0.142	-0.416	0.678	[-0.338, 0.22]
Negative Affect	-0.295	0.356	-0.829	0.393	[-0.991, 0.402]
Hunger Rating	-0.001	0.004	-0.288	0.773	[-0.009, 0.006]
EB (external)	0.399	0.386	1.032	0.302	[-0.359, 1.156]
EB (emotional)	-0.33	0.259	-1.273	0.203	[-0.838, 0.178]
EB (restrictive)	0.346	0.259	1.336	0.181	[-0.161, 0.853]

	Random Effects			
	Variance	SD	Correlation	
(Intercept)	2.02	1.421		
condition (hungry)	0.825	0.908	-0.32	
DT (delay attribute)	0.092	0.304	-0.38	-0.05

Note. Results of the GLMM impatient choice ~ condition + DT (delay option) + DT (delay attribute) + gender + BMI + age + Positive Affect + Negative Affect + Hunger Rating + EB (external) + EB (emotional) + EB (restictive) + (1+condition|subject), using binomial distribution with the logit link function and optimizing fit with bobyqa, 18375 observations in 53 participants, *AIC*=14899.7; DT (delay option) refers to the relative scaled dwell time on the sooner smaller vs later larger option; DT (delay attribute) refers to the relative scaled dwell time on the attribute for delay versus amount; given the large variation between participants with respect to the effect of DT (delay attribute) (see **Figure S6**), we account for this using a random slope, which also improved AIC; BMI and age were centralized; for Positive and Negative Affect as well as Hunger Rating we used the change score (t3-t0); EB refers to the eating behavior questionnaire; *SE*=standard error, *CI*=confidence interval calculated using Wald approximation, *SD*=standard deviation.

Table S8

Effect of hunger on RTs in intertemporal discounting task

	Fixed Effects				
	coefficient	SE	z-statistic	p-value	CI
(Intercept)	1.998	0.159	12.569	<0.001	[1.764, 2. 29]
condition (hungry)	0.05	0.122	0.407	0.684	[-0.155, 0.233]
delay VD	-0.032	0.001	23.505	<0.001	[-0.035, -0.03]
amount VD	-0.139	0.001	-106.004	<0.001	[-0.141, -0.136]
condition x delay VD	-0.007	0.001	-5.112	<0.001	[-0.01, -0.004]
condition x amount VD	-0.004	0.001	-3.117	0.002	[-0.007, -0.002]

	Random Effects		
	Variance	SD	Correlation
(Intercept)	0.516	0.718	
condition (hungry)	0.321	0.567	-0.59
Residual	0.285	0.534	

Note. Results of the GLMM RT ~ condition * (delay VD + amount VD) + (1+condition|subject), using a Gamma distribution with the identity link function and optimizing fit with bobyqa, 24611 observations in 70 participants, the model indicated a convergence warning; however, an allFit analysis confirmed that fixed and random effects estimates and logliklihoods were consistent across converging optimisers, indicating the reliability of the results; we used the absolute scaled delay and amount value difference

(VD); *AIC*=58953.7; *SE*=standard error, *CI*=confidence interval calculated using Wald approximation, *SD*=standard deviation.

Figure S5

Variation in the effect of relative attribute DT on social choice

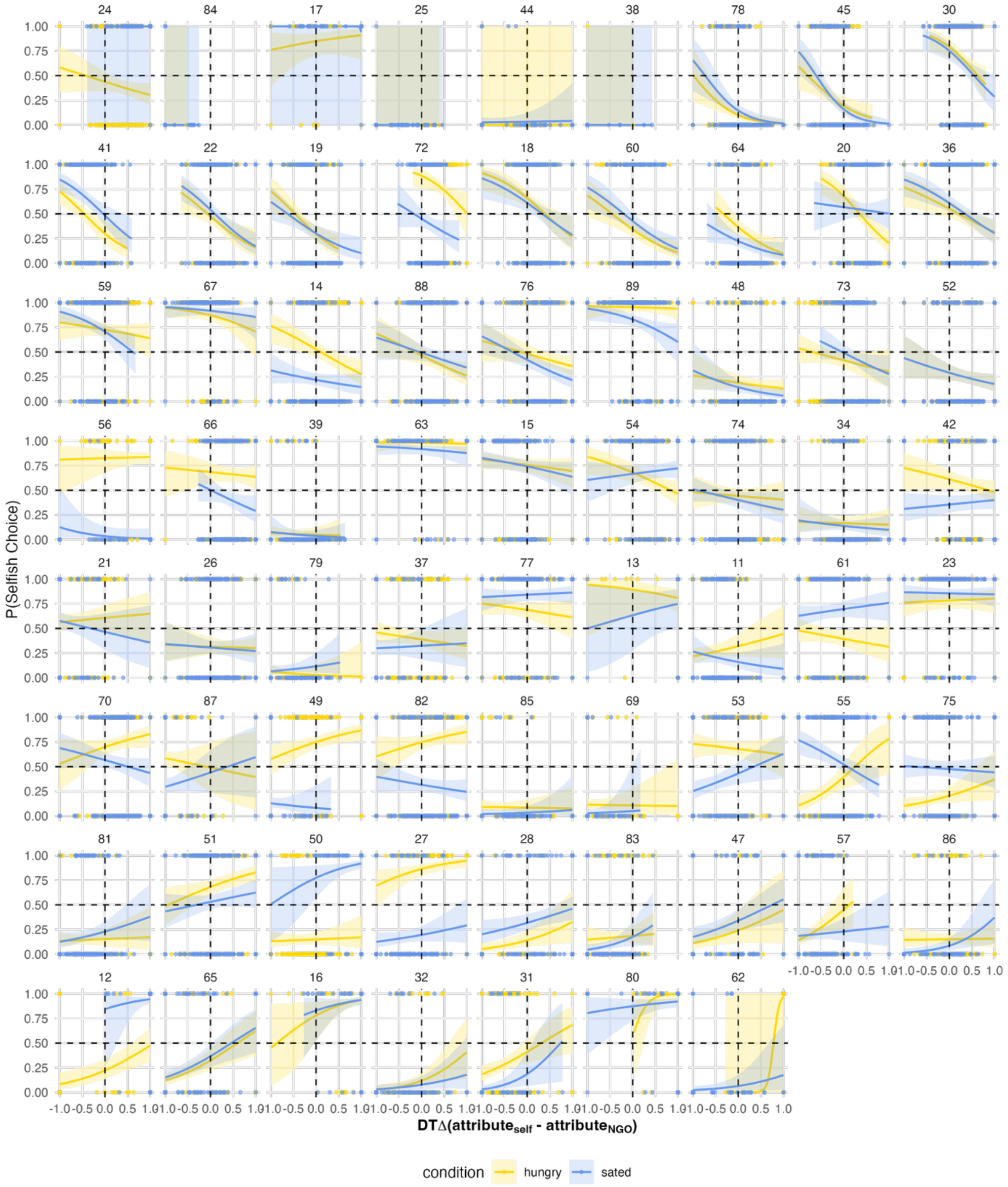

Note. Each subplot represents the effect of relative DT (self – NGO) on selfish choice of one participant, in each condition (yellow = hungry; blue = sated), in some participants looking longer at self, decreased the probability of selfish choice (rows 1-3); in some participants looking longer at self, did not affect selfish choice (rows 4-5.5); in some participants looking longer at self, increased the probability of selfish choice (rows 5.5-7).

Table S9

Effect of hunger on social choice

	Fixed Effects				
	coefficient	SE	z-statistic	p-value *(cor. p-value)*	CI
(Intercept)	-0.743	0.286	-2.599	0.009	[-1.304, -0.183]
condition (hungry)	-0.172	0.163	-1.051	0.294 *(0.333)*	[-0.162, 0.513]
DT (selfish option)	1.465	0.026	56.343	<0.001	[1.218, 1.304]
DT (selfish attribute)	-0.138	0.08	-1.726	0.084	[-0.216, -0.027]

	Random Effects			
	Variance	SD	Correlation	
(Intercept)	5.635	2.374		
condition (hungry)	1.875	1.369	-0.27	
DT (selfish attribute)	0.122	0.35	-0.14	0.20

Note. Results of the GLMM selfish choice ~ condition + DT (selfish option) + DT (selfish attribute) + (1 + condition + DT (selfish attribute) | subject), using binomial distribution with the logit link function and optimizing fit with bobyqa, 23971 observations in 70 participants, AIC=18851; DT (selfish option) refers to the relative scaled dwell time on the selfish option; DT (selfish attribute) refers to the relative scaled dwell time on the selfish attribute given the large variation between participants with respect to the effect of DT on the attribute for self (see **Figure S3**), we account for this using a random slope; SE=standard error, we applied Bonferroni correction for multiple comparisons for corrected p-value in parentheses and italic, CI=confidence interval calculated using Wald approximation, SD=standard deviation.

Table S10

Effect of hunger rating on social choice

	Fixed Effects			
	coefficient	SE	z-statistic	p-value *(cor. p-value)*
(Intercept)	-0.626	0.28	-2.237	0.025
delta hunger	-0.003	0.003	-0.954	0.34 *(1)*
DT (selfish option)	1.266	0.023	55.812	<0.001
DT (selfish attribute)	-0.136	0.049	-2.78	0.005

	Random Effects			
	Variance	SD	Correlation	
(Intercept)	5.716	2.391		
condition (hungry)	1.962	1.401	-0.28	
DT (selfish attribute)	0.124	0.353	-0.16	0.22

Note. Results of the GLMM selfish choice ~ delta hunger + DT (selfish option) + DT (selfish attribute) + (1 + condition + DT (selfish attribute) | subject), using binomial distribution with the logit link function and optimizing fit with bobyqa, 23971 observations in 70 participants, AIC=18851; delta hunger, refers to the difference in hunger ratings between two time points (sated: before protein shake – after protein shake administration; hungry: at lab arrival – before start of choice task); DT (selfish option) refers to the relative scaled dwell time on the selfish option; DT (selfish attribute) refers to the relative scaled dwell time on the selfish attribute given the large variation between participants with respect to the effect of DT on the attribute for self, we account for this using a random slope; SE=standard error, we applied Bonferroni correction for multiple comparisons for corrected p-value in parentheses and italic, SD=standard deviation.

Table S11

Effect of hunger state, attention, mood and demographics on social choice

	Fixed Effects				
	coefficient	SE	z-statistic	p-value	CI
(Intercept)	1.852	1.762	1.052	0.293	[-1.6, 5.305]
condition (hungry)	-0.606	0.38	-1.594	0.111	[-1.351, 0.139]
DT (selfish option)	1.268	0.026	49.451	<0.001	[1.218, 1.318]
DT (selfish attribute)	-0.13	0.057	-2.281	0.023	[-0.241, -0.018]
gender	-0.737	0.709	-1.04	0.298	[-2.126, 0.652]
BMI	0.057	0.066	0.865	0.387	[-0.072, 0.185]
age	-0.082	0.1704	-2.04	0.041	[-0.161, -0.003]
Positive Affect	0.04	0.209	2.193	0.847	[-0.369, 0.45]
Negative Affect	0.088	0.551	0.159	0.874	[-0.993, 0.169]
Hunger Rating	0.013	0.006	2.081	0.037	[0.001, 0.024]
EB (external)	0.166	0.639	0.26	0.795	[-1.087, 1.419]
EB (emotional)	-0.724	0.426	-1.701	0.089	[-1.558, 0.11]
EB (restrictive)	-0.059	0.416	-0.141	0.888	[-0.875, 0.757]

	Random Effects			
	Variance	SD	Correlation	
(Intercept)	5.381	2.32		
condition (hungry)	1.995	1.413	-0.49	
DT (selfish attribute)	0.13	0.36	-0.32	0.27

Note. Results of the GLMM selfish choice ~ condition + DT (selfish option) + DT (selfish attribute) + gender + BMI + age + Positive Affect + Negative Affect + Hunger Rating + EB (external) + EB (emotional) + EB (restictive) + (1+condition+ DT (selfish attribute)|subject), using binomial distribution with the logit link function and optimizing fit with bobyqa, 17660 observations in 53 participants, *AIC*=4403.5; DT (selfish option) refers to the relative scaled dwell time on the selfish versus prosocial option; DT (selfish attribute) refers to the relative scaled dwell time on the selfish versus NGO attribute; given the large variation between participants with respect to the effect of DT on the attribute for self (see **Figure S4**), we account for this using a random slope, which also improved AIC; BMI and age were centralized; for Positive and Negative Affect as well as Hunger Rating we used the scaled change score (t3-t0); EB refers to the eating behavior questionnaire, and scores were z-scored; SE=standard error, CI=confidence interval calculated using Wald approximation, SD =standard deviation.

Table S12

Effect of hunger on RTs in social choice task

	Fixed Effects				
	coefficient	SE	z-statistic	p-value	CI
(Intercept)	2.027	0.134	15.082	<0.001	[1.764, 2.29]
condition (hungry)	0.029	0.099	0.393	0.694	[-0.155, 0.233]
self VD	-0.024	0.006	-3.976	<0.001	[-0.036, -0.012]
NGO VD	-0.082	0.006	-14.372	<0.001	[-0.093, -0.071]
condition x self VD	-0.002	0.009	-0.243	0.808	[-0.019, -0.015]
condition x NGO VD	-0.013	0.008	-1.539	0.124	[-0.004, 0.029]

	Random Effects		
	Variance	SD	Correlation
(Intercept)	0.388	0.623	
condition (hungry)	0.227	0.477	-0.56
Residual	0.295	0.543	

Note. Results of the GLMM RT ~ condition * (self VD + NGO VD) + (1+condition|subject), using a Gamma distribution with the identity link function and optimizing fit with bobyqa, 23971 observations in 70 participants, we used the absolute scaled self and NGO value difference; *AIC*=58139.7; SE=standard error, CI=confidence interval calculated using Wald approximation, SD=standard deviation.

Figure S6

Individual differences in social choice

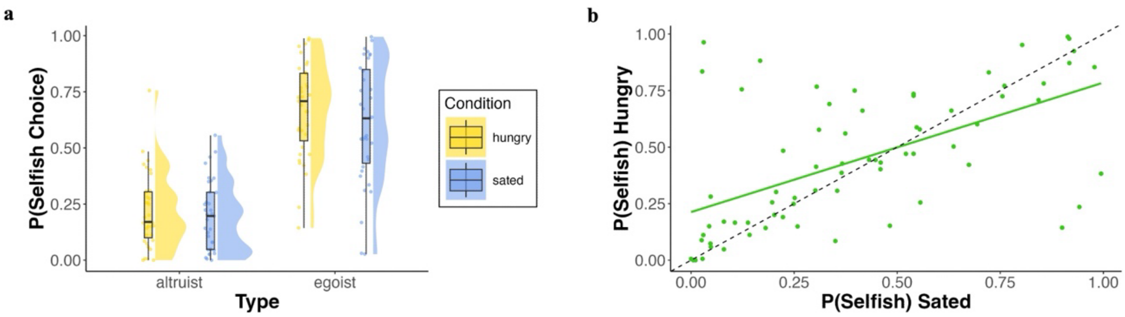

Note. **a)** Proportion of selfish choices by idiosyncratic preference and condition. We used a median split to categorize participants to possess either selfish (egoist) or prosocial (altruist) tendencies. While there is a difference in proportion of selfish choices between types in both hungry ($t(64.364)=9.974$, $p<0.001$, $d=1.243$) and sated condition ($t(54.356)=7.925$, $p<0.001$, $d=1.075$), there is no difference between condition: participants with selfish preference in the sated condition do not change their choice patterns when hungry ($t(34)=0.996$, $p<0.327$, $d=0.17$) and participants with prosocial preference in the sated condition do not change their choice patterns when hungry ($t(34)=0.742$, $p<0.463$, $d=0.127$) **b)** Correlation of selfish choices between conditions ($r= 0.581$, $t(68)=5.888$, $p<0.001$, $d=0.714$) indicating that choice patterns remained stable between conditions.

Table S13

Mediation coefficients

	Mean	SE	Median	2.50%	97.50%	n_eff	Rhat
a	0.056	0.03	0.06	0.003	0.11	45676	1
b	1.1	0.8	1.1	0.94	1.3	20768	1
cp	0.19	0.11	0.19	-0.02	0.4	29129	1
me	0.08	0.04	0.08	0.02	0.15	45354	1
c	0.27	0.12	0.27	0.03	0.52	27964	1
pme	0.36	4.08	0.3	0.06	1.05	97291	1

Note. a is the effect of hunger state on the scaled DT proportion difference between tasty and healthy option (i.e., "attention"); *b* is the effect of attention on food choice; *cp* refers to the indirect effect of hunger state on food choice taking attention into account; *c* is the direct effect of hunger state on food choice, when attention is not considered; *me* refers to the mediation effect, thus the combination of paths *a* and *b*; *pme* refers to the proportion of the effect that is mediated. Output refers to posterior mean, standard error (*SE*), median, and credible interval (*CI*); *n_eff* is the number of effective posterior samples, to obtain confident estimates, in line with Vuorre and Bolger (2018) it should be larger than 100; *Rhat* is the scale reduction factor, to accurately predict posterior distributions, it should be 1, but values smaller than 1.05 are acceptable [3]; we used 4 chains and set the number of iterations to 50000 with a warmup of 25000, resulting in 100000 effective samples.

Table S14

Standard deviations of subject-level effects (random effects), their covariances and correlations

	Mean	SE	Median	2.50%	97.50%	n_eff	Rhat
tau_a	0.15	0.03	0.15	0.09	0.21	8155	1
tau_b	0.65	0.07	0.65	0.53	0.8	5933	1
tau_cp	0.78	0.09	0.78	0.62	0.97	38589	1
covab	0.02	0.02	0.02	-0.01	0.06	52592	1
corrab	0.21	0.17	0.22	-0.13	0.53	56374	1

Figure S7

Proportion of dwell time on hot attributes between conditions and across tasks

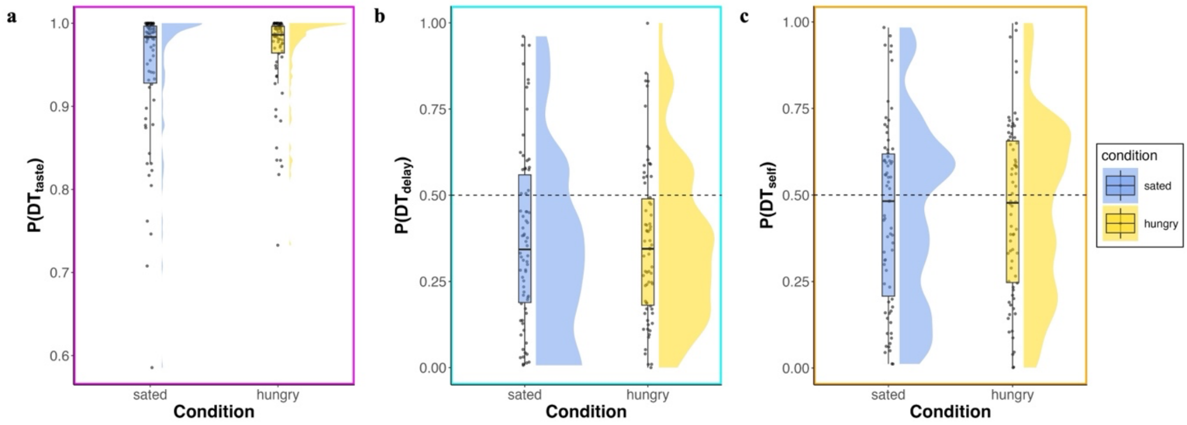

Note. **a)** hungry participants are more likely to look at food images (i.e., taste attribute) ($t(69)= 2.598$, $p=.012$, $d=0.312$) **b)** no difference between conditions with respect to DT on delay attribute ($t(69)= -0.846$, $p=.4$, $d=-0.102$); participants are less likely to look at the attribute for delay in both hungry ($t(69)= -5.181$, $p<.001$, $d=0.36$) and sated condition($t(69)= -4.158$, , $p<.001$, $d=0.374$) ; **c)** no difference between conditions with respect to DT on self attribute ($t(69)= 0.395$, $p=.694$, $d=0.048$).

Figure S8

Fixation pattern across tasks

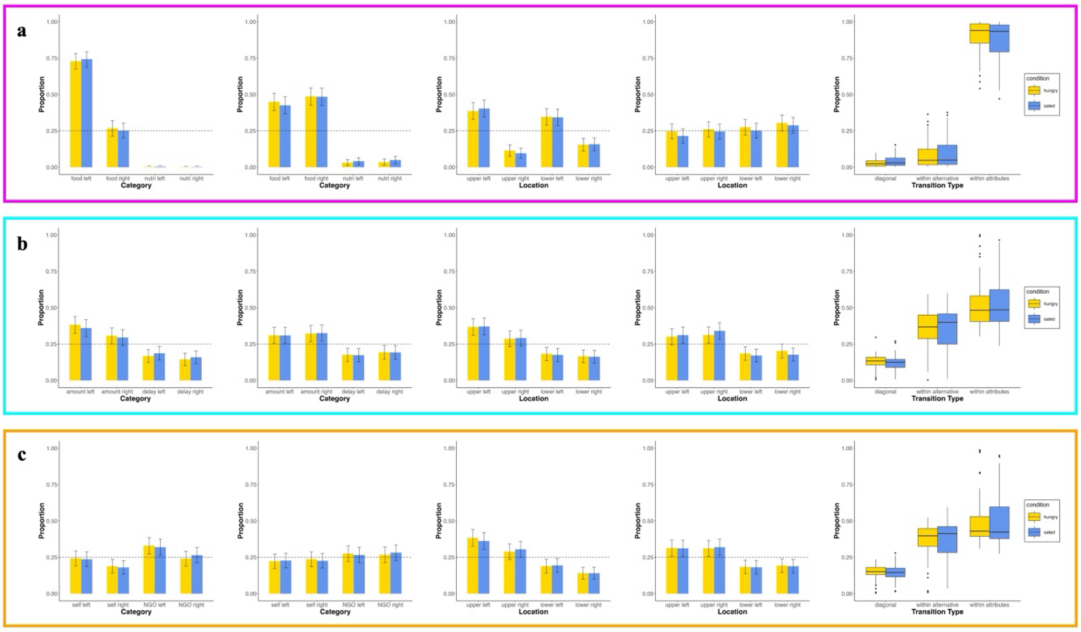

Note. From left to right first and last fixation by category and location as well as transition patterns as obtained through Payne Index [4] for **a)** food, **b)** intertemporal and **c)** social choice tasks.

Table S15

Parameter estimation: prior distributions

	Parameters							
	α	nDT	d	β	ω	θ	ϕ_{hot}	ϕ_{cold}
$Group\ mean_{baseline}$	N(2,1)	N(-1,1)	N(0,0.5)	N(0,0.5)	N(0,0.5)	N(0,0.5)	N(0,0.5)	N(0,0.5)
$Group\ SD_{baseline}$	HN(0,3)	HN(0,1)	HN(0,0.5)	HN(0,0.5)	HN(0,0.5)	HN(0,0.5)	HN(0,0.5)	HN(0,0.5)
$Group\ mean_{change}$	N(0,1)	N(0,1)	N(0,0.25)	N(0,0.25)	N(0,0.25)	N(0,0.5)	N(0,0.5)	N(0,0.25)
$Group\ SD_{change}$	HN(0,1)	HN(0,1)	HN(0,0.25)	HN(0,0.25)	HN(0,0.25)	HN(0,0.25)	HN(0,0.25)	HN(0,0.25)

Note. To test differences between hunger states, we estimated baseline parameters for the sated condition and a change score for the hungry condition (i.e., $parameter_{hungry}$ = $parameter_{sated}$ + change score). We implemented a hierarchical Bayesian model [5], in JAGS and R using the R2jags package [6]. Group-level parameters were drawn from a normal distribution and half-normal distribution [7]. Individual parameters were drawn from N(μ_{Group}, SD_{Group}). We used a soft-plus transformation for α, nDT and d and phi transformations for β and ω. To speed up model-fit we used piecewise constant averaging [8]. Overall, we used eight chains, 60000 iterations, 30000 burnin samples and a thinning of 12. We used Rhat to assess convergence with a threshold of 1.05 [9] and quantified model fit using deviance information criterion (DIC, Spiegelhalter et al., 2002); α = boundary separation; nDT = non-decision time; d = drift scaling; β = relative starting point bias; relative hot decision weight (i.e., taste, delay, self); θ = theta (i.e., attentional discounting of unattended option); ϕ_{hot} = phi hot (i.e., attentional discounting of unattended hot attribute); ϕ_{cold} = phi cold (i.e., attentional discounting of unattended cold attribute).

Figure S9

Parameter Estimates of the maaDDM2ϕ across choice tasks

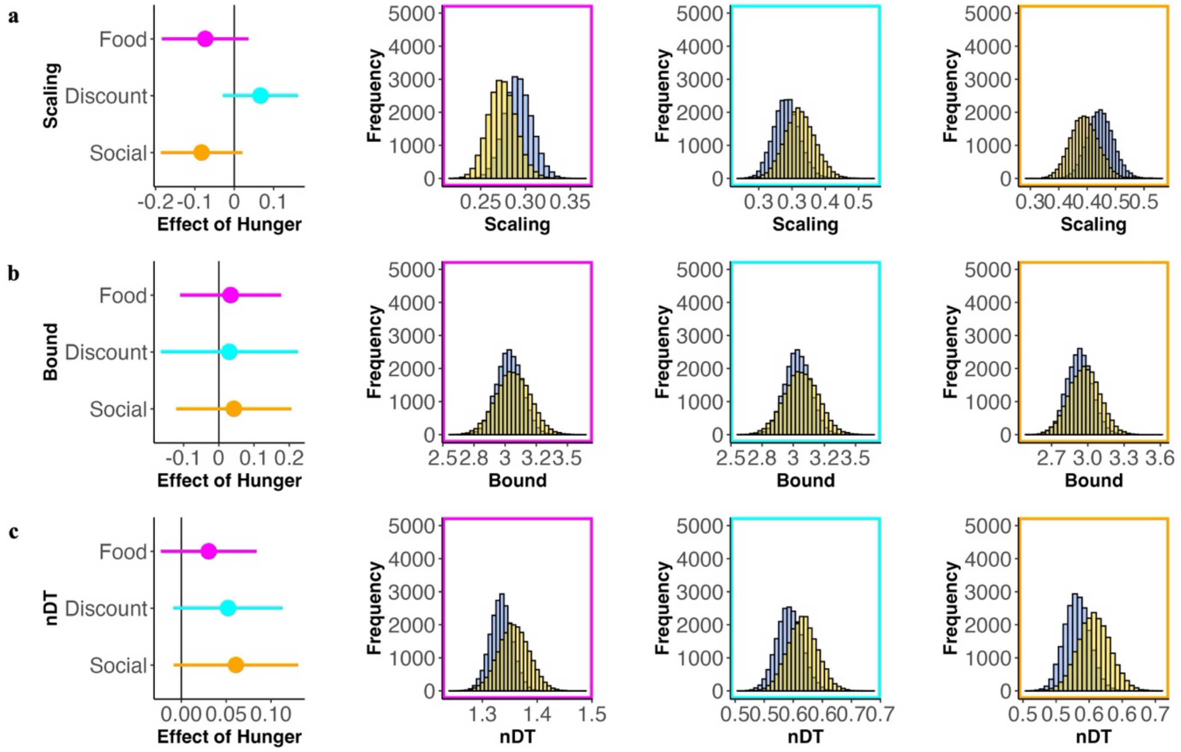

Note. Effect of hunger state on group-level parameter estimates of the maaDDM2ϕ across tasks (pink=food; turquoise=intertemporal discounting; orange=social) on the left and the distributions per condition (blue=sated; yellow=hungry) on the right; no effect of hunger state on **a)** drift scaling, **b)** boundary separation and **c)** non-decision time across tasks and conditions.

Figure S10

Parameter estimation of hyperbolic model

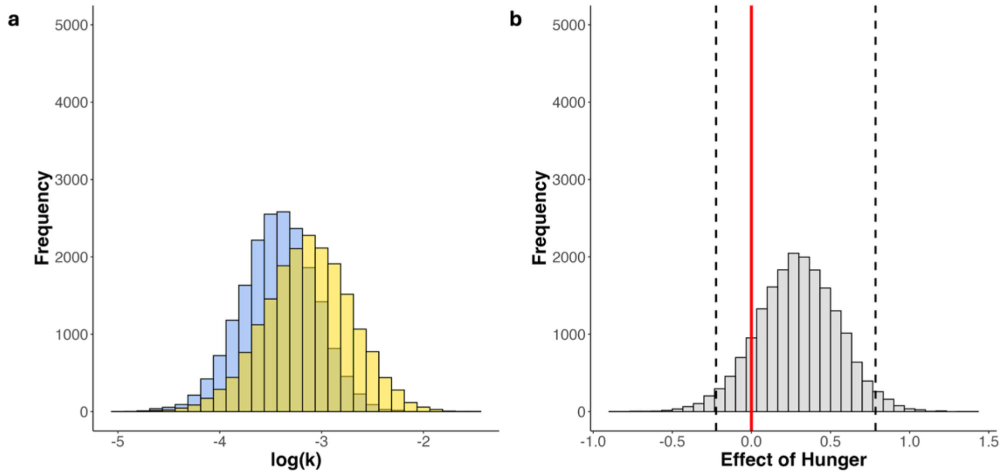

Note. Each participant's baseline $\log(\kappa)$ was drawn from a group-level normal distribution $N(\mu_{\log(\kappa)}, \sigma_{\log(\kappa)})$. Similar to the hunger effect modeled in our DDMs, we implemented subject-specific random slopes in this model, with normally distributed mean group shift with standard deviation to capture the individual variability in the shift. Similarly, we modelled the inverse temperature β with a group-level baseline and a fixed condition shift. As in our DDMs, amount and delay were rescaled to a scale form 1-10. Following the recommendation of Vincent (2016), we

specified weakly informative priors: $\mu_{\log(\kappa)}$ ~N(-3, 10), $\mu_{\log(\beta)}$ ~N(0, 10) and gamma distributions for the precision parameters $Gamma$(0.001, 0.001). The effect of hunger state was centered at 0 with N(0,1) for both κ and β. The model was estimated with four MCMC chains of 60000 iterations each and 20000 burn-in and a thinning factor of 12. **a)** posterior distributions of κ for hungry (yellow) and sated (blue) conditions respectively, **b)** the posterior distribution of the effect of hunger. The HDI includes 0 indicating no credible differences in discounting between hunger states.

Figure S11

Parameter recovery of the food choice task

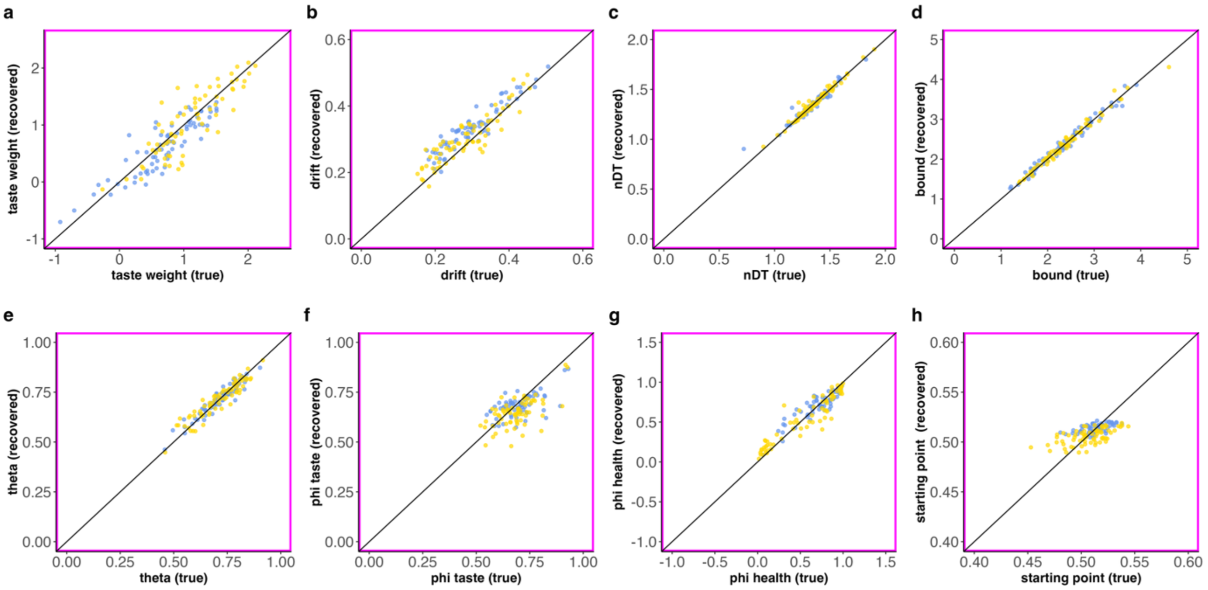

Note. We simulated data for each participant in each condition based on the means of each parameter, empirical taste and health ratings and fixation patterns; **a)** the correlation between the true and recovered relative taste weight was r_{sated}=0.892 and r_{hungry}=0.907; **b)** the correlation between the true and recovered drift scaling was r_{sated}=0.925 and r_{hungry}=0.893; **c)** the correlation between the true and recovered non-decision time was r_{sated}=0.968 and r_{hungry}=0.983; **d)** the correlation between the true and recovered boundary separation was r_{sated}=0.988 and r_{hungry}=0.99; **e)** the correlation between the true and recovered theta was r_{sated}=0.947 and r_{hungry}=0.939; **f)** the correlation between the true and recovered phi hot was r_{sated}=0.65 and r_{hungry}=0.658; **g)** the correlation between the true and recovered phi cold was r_{sated}=0.921 and r_{hungry}=0.945; **f)** the correlation between the true and recovered relative starting point bias was r_{sated}=0.567 and r_{hungry}=0.53.

Figure S12

Parameter recovery of the intertemporal discounting task

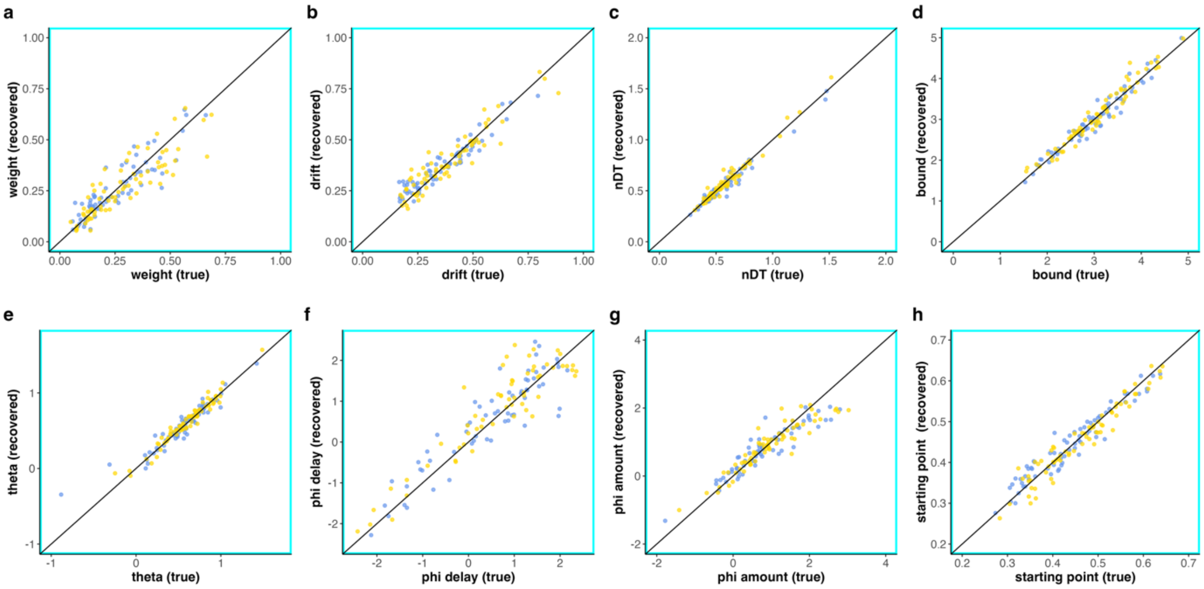

Note. We simulated data for each participant in each condition based on the means of each parameter, empirical delay and amount values and fixation patterns; **a)** the correlation between the true and recovered relative delay weight was r_{sated}=0.896 and r_{hungry}=0.89; **b)** the correlation between the true and recovered drift scaling was r_{sated}=0.938 and r_{hungry}=0.923; **c)** the correlation between the true and recovered non-decision time was r_{sated}=0.978 and r_{hungry}=0.978; **d)** the correlation between the true and recovered boundary separation was r_{sated}=0.982 and r_{hungry}=0.979; **e)** the correlation between the true and recovered theta was r_{sated}=0.948 and r_{hungry}=0.958; **f)** the correlation between the true and recovered phi hot was r_{sated}=0.927 and r_{hungry}=0.909; **g)** the correlation between the true and recovered phi cold was r_{sated}=0.941 and r_{hungry}=0.937; **f)** the correlation between the true and recovered relative starting point bias was r_{sated}=0.963 and r_{hungry}=0.965.

Figure S13

Parameter recovery of the social choice task

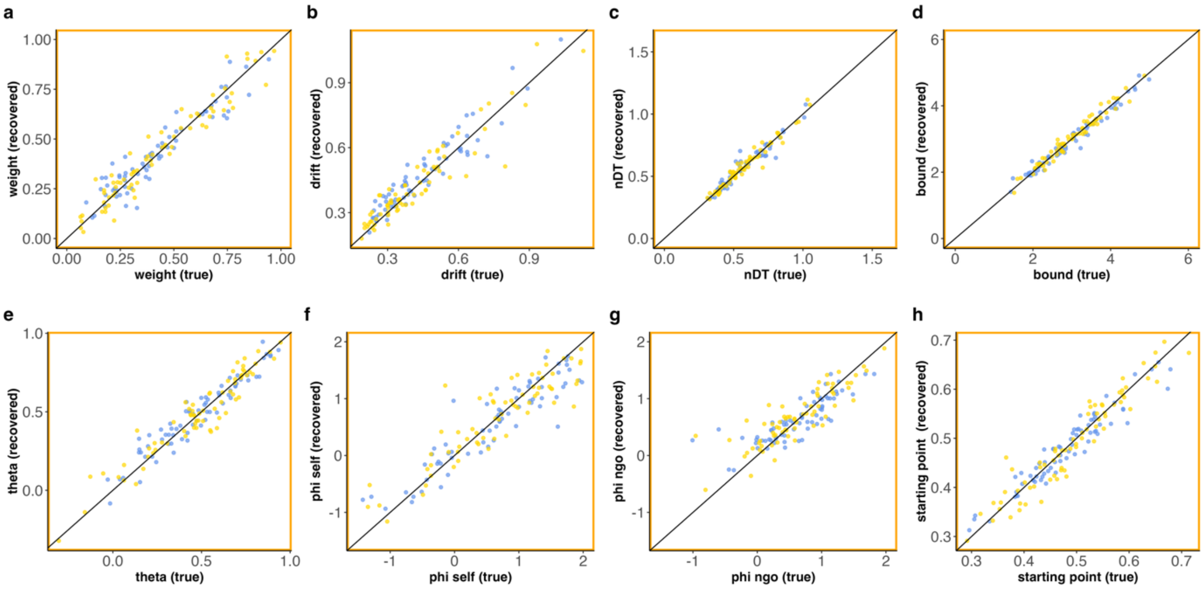

Note. We simulated data for each participant in each condition based on the means of each parameter, empirical self and NGO values and fixation patterns; **a)** the correlation between the true and recovered relative self weight was r_{sated}=0.934 and r_{hungry}=0.951; **b)** the correlation between the true and recovered drift scaling was r_{sated}=0.945 and r_{hungry}=0.939; **c)** the correlation between the true and

recovered non-decision time was r_{sated}=0.97 and r_{hungry}=0.98; **d)** the correlation between the true and recovered boundary separation was r_{sated}=0.986 and r_{hungry}=0.983; **e)** the correlation between the true and recovered theta was r_{sated}=0.958 and r_{hungry}=0.951; **f)** the correlation between the true and recovered phi hot was r_{sated}=0.925 and r_{hungry}=0.902; **g)** the correlation between the true and recovered phi cold was r_{sated}=0.834 and r_{hungry}=0.873; **f)** the correlation between the true and recovered relative starting point bias was r_{sated}=0.959 and r_{hungry}=0.954.

References

1. March, J. & Gluth, S. The Hungry Lens: Hunger Shifts Attention and Attribute Weighting in Dietary Choice. *eLife* **13**, (2024).
2. March, J. & Gluth, S. Hunger shifts attention and attribute weighting in dietary choice. *eLife* **13**, RP103736 (2025).
3. Vuorre, M. & Bolger, N. Within-subject mediation analysis for experimental data in cognitive psychology and neuroscience. *Behav. Res. Methods* **50**, 2125–2143 (2018).
4. Payne, J. W. Task complexity and contingent processing in decision making: An information search and protocol analysis. *Organ. Behav. Hum. Perform.* **16**, 366–387 (1976).
5. Farrell, S. & Lewandowsky, S. *Computational Modeling of Cognition and Behavior.* (Cambridge University Press, Cambridge, 2018). doi:10.1017/CBO9781316272503.
6. Su, Y.-S. & Yajima, M. R2jags: Using R to Run 'JAGS'. 0.8-9 https://doi.org/10.32614/CRAN.package.R2jags (2021).
7. Kraemer, P. M. & Gluth, S. Episodic Memory Retrieval Affects the Onset and Dynamics of Evidence Accumulation during Value-based Decisions. *J. Cogn. Neurosci.* **35**, 692–714 (2023).
8. Lombardi, G. & Hare, T. Piecewise constant averaging methods allow for fast and accurate hierarchical Bayesian estimation of drift diffusion models with time-varying evidence accumulation rates. Preprint at https://doi.org/10.31234/osf.io/5azyx (2021).

9. Gelman, A. & Rubin, D. B. Inference from Iterative Simulation Using Multiple Sequences. *Stat. Sci.* **7**, 457–472 (1992).

10. Spiegelhalter, D. J., Best, N. G., Carlin, B. P. & Van Der Linde, A. Bayesian measures of model complexity and fit. *J. R. Stat. Soc. Ser. B Stat. Methodol.* **64**, 583–639 (2002).rences
